# Aminoglycoside Antibiotics Inhibit Phage Infection by Blocking an Early Step of the Infection Cycle

**DOI:** 10.1128/mbio.00783-22

**Published:** 2022-05-04

**Authors:** Larissa Kever, Aël Hardy, Tom Luthe, Max Hünnefeld, Cornelia Gätgens, Lars Milke, Johanna Wiechert, Johannes Wittmann, Cristina Moraru, Jan Marienhagen, Julia Frunzke

**Affiliations:** a Institute of Bio- und Geosciences, IBG-1: Biotechnology, Forschungszentrum Jülich, Jülich, Germany; b Leibniz Institute DSMZ—German Collection of Microorganisms and Cell Cultures, Braunschweig, Germany; c Institute for Chemistry and Biology of the Marine Environment, Carl von Ossietzky University Oldenburg, Oldenburg, Germany; d Institute of Biotechnology, RWTH Aachen University, Aachen, Germany; National Institute of Child Health and Human Development

**Keywords:** *Streptomyces*, aminoglycosides, antibiotics, bacteriophages, phage defense, phage-host interaction

## Abstract

In response to viral predation, bacteria have evolved a wide range of defense mechanisms, which rely mostly on proteins acting at the cellular level. Here, we show that aminoglycosides, a well-known class of antibiotics produced by *Streptomyces*, are potent inhibitors of phage infection in widely divergent bacterial hosts. We demonstrate that aminoglycosides block an early step of the viral life cycle, prior to genome replication. Phage inhibition was also achieved using supernatants from natural aminoglycoside producers, indicating a broad physiological significance of the antiviral properties of aminoglycosides. Strikingly, we show that acetylation of the aminoglycoside antibiotic apramycin abolishes its antibacterial effect but retains its antiviral properties. Altogether, our study expands the knowledge of aminoglycoside functions, suggesting that aminoglycosides not only are used by their producers as toxic molecules against their bacterial competitors but also could provide protection against the threat of phage predation at the community level.

## INTRODUCTION

Bacteriophages are viruses that prey upon bacteria. Facing the existential threat posed by phage predation, prokaryotes have developed numerous lines of defense, which together form the prokaryotic “immune system” (1). In response, phages have evolved a multitude of ways to circumvent these barriers, thereby fostering the diversification of bacterial antiviral strategies. Recent bioinformatics-guided screenings revealed a large number of previously unknown antiviral defense systems (2, 3). However, the majority of currently known prokaryotic defense systems rely on a wide range of molecular mechanisms but are mediated mainly by protein or RNA complexes (4).

Environmental bacteria produce a wide range of small molecules, conferring producer cells a specific fitness advantage in competitive or predatory interactions. However, the potential antiphage role of this extensive chemical repertoire remains largely unexplored. Recently, new types of defense systems that rely on small molecules rather than on proteins or RNA have been discovered (5, 6). Anthracyclines are secondary metabolites naturally produced by *Streptomyces* species and were shown to inhibit infection by double-stranded-DNA (dsDNA) phages (5). These molecules act as DNA-intercalating agents and block the replication of phage—but not bacterial—DNA. Since these secondary metabolites are excreted by *Streptomyces* cells and are diffusible molecules, their production may provide broad protection against dsDNA phages at the community level.

In nature, producers of secondary metabolites are generally resistant to the molecules they synthesize (7, 8). This feature is of special importance when screening small molecules for antiviral properties, as toxic effects on bacterial growth would prevent the appreciation of any inhibition of phage infection. In this study, we leveraged this principle to look for phage inhibition by secondary metabolites, using bacterial hosts resistant to the compounds tested.

Aminoglycosides are antibiotics well known for their bactericidal effect by targeting the 30S subunit of the ribosome and thereby either directly inhibiting protein synthesis or, for most aminoglycosides, promoting mistranslation. The aminoglycoside streptomycin, discovered in 1943, was the first antibiotic active against Mycobacterium tuberculosis (9). Strikingly, we observed strong phage inhibition in the presence of aminoglycosides when using strains resistant to the antibiotic. In agreement with this observation, decades-old reports described the inhibition of various phages by streptomycin (10–12). However, the biological significance of these observations was not explored, and the underlying mechanism of action remains unclear. For these reasons, we focused our efforts on aminoglycosides and set out to investigate their potential antiphage properties.

In this study, we show that aminoglycoside antibiotics inhibit phages infecting the actinobacterial model species Streptomyces venezuelae and Corynebacterium glutamicum as well as the λ phage infecting Escherichia coli. Investigations of the mechanism of action point toward a blockage of phage infection occurring after DNA injection but before genome replication. Furthermore, the antiphage activity observed with the purified aminoglycoside apramycin could be reproduced with supernatants from the natural producer Streptoalloteichus tenebrarius, suggesting a broad physiological significance of the antiphage properties of aminoglycosides.

## RESULTS

### Aminoglycosides inhibit a broad range of phages.

**FIG 1:**
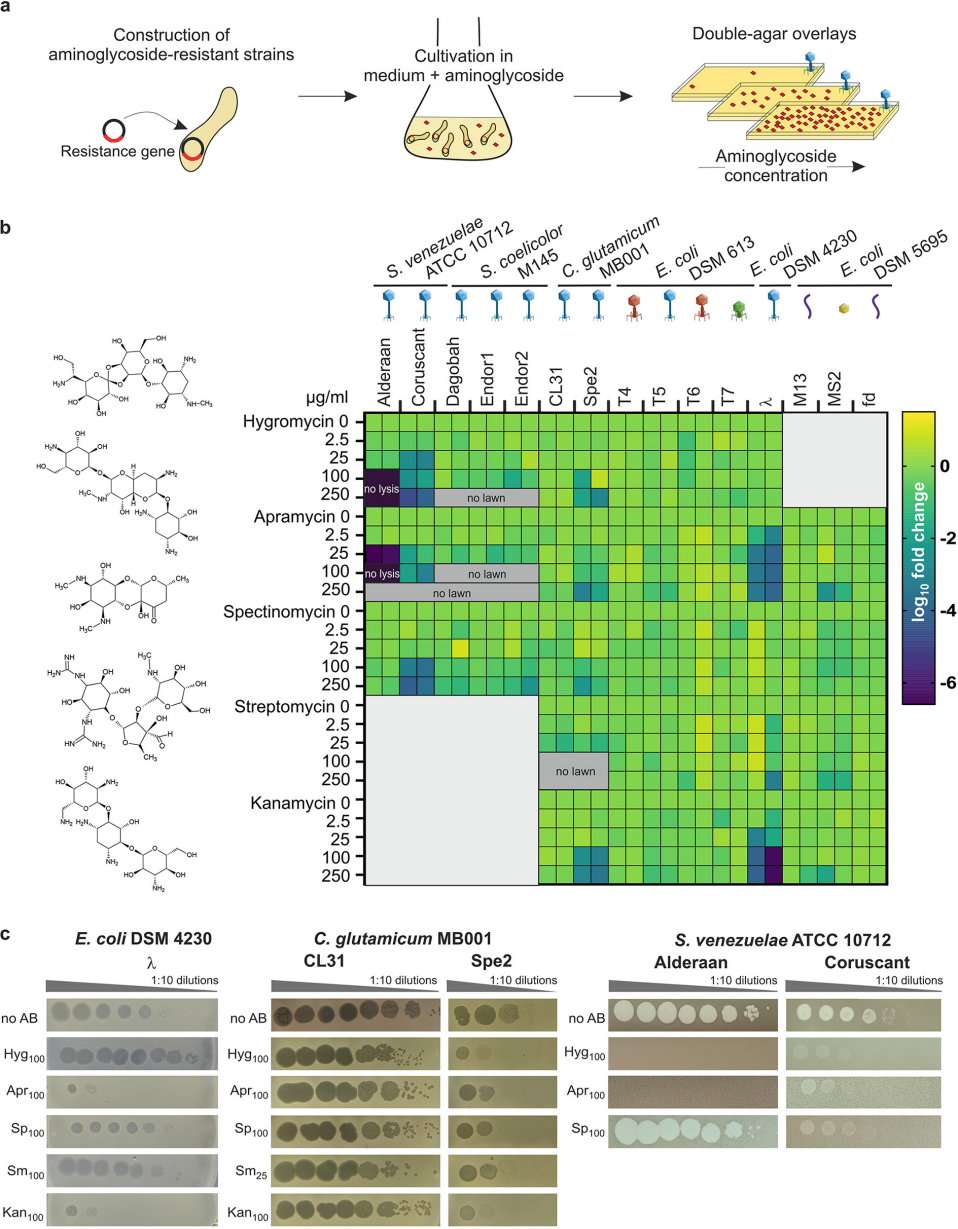
Aminoglycosides inhibit a wide range of phages. (a) Schematic representation of the screening for the antiphage effect of different aminoglycosides. Strains resistant to the aminoglycosides were constructed using plasmid-borne resistance cassettes and subsequently challenged by phages in the presence of increasing aminoglycoside concentrations. (b) Overview of the screening results, showing the log_10_ fold change in plaque formation by tested phages relative to the aminoglycoside-free control. Molecular structures of the aminoglycosides tested are indicated on the left. High concentrations of aminoglycosides prevented in some cases either the formation of plaque or lysis zone by the spotted phages (“no lysis”) or bacterial growth (“no lawn”). *n* = 2 independent biological replicates. The different phage morphologies are depicted with icons according to the following color scheme: blue, *Siphoviridae*; red, *Myoviridae*; green, *Podoviridae*; purple, *Inoviridae*; yellow, *Leviviridae*. (c) Exemplary pictures from propagation assays performed in the presence of the indicated aminoglycoside concentration. Results are representative of two biological replicates.

To investigate a potential antiviral activity of aminoglycosides, we first constructed resistant strains carrying a plasmid-borne resistance cassette encoding an aminoglycoside-modifying enzyme (Table S1 and S2A). With respect to the aminoglycosides selected for this study, we focused on antibiotics produced by *Streptomyces* species and included the atypical aminoglycoside streptomycin, aminoglycosides containing a monosubstituted deoxystreptamine ring (apramycin and hygromycin), kanamycin (4,6-di-substituted deoxystreptamine ring), and the aminocyclitol spectinomycin (13, 14). We challenged the aminoglycoside-resistant strains with a set of different phages using double-agar overlays with increasing aminoglycoside concentrations as screening platform (Fig. 1a). In the screening, we included phages from three different viral realms (15): dsDNA viruses from the order *Caudovirales* in *Duplodnaviria* (families *Sipho*-, *Myo*-, and *Podoviridae*), single-stranded DNA (ssDNA) viruses from the family *Inoviridae* in *Monodnaviria*, and ssRNA viruses from the family *Leviviridae* in *Riboviria* (Table S2B). The efficiency of plating comparing plaque formation under aminoglycoside pressure with aminoglycoside-free conditions was calculated for phages infecting either the actinobacterial model species Streptomyces venezuelae, Streptomyces coelicolor, and Corynebacterium glutamicum or the Gram-negative species Escherichia coli (Fig. 1b).

10.1128/mbio.00783-22.1 TABLE S1 Aminoglycoside-modifying enzymes used in this study. Download Table S1, DOCX file, 0.01 MB. Copyright © 2022 Kever et al. 2022 Kever et al. https://creativecommons.org/licenses/by/4.0/ This content is distributed under the terms of the Creative Commons Attribution 4.0 International license.

10.1128/mbio.00783-22.2 TABLE S2 (A) Bacterial strains used in this study. (B) Phages used in this study. (C) Plasmids used in this study. Insert DNA was amplified using the listed oligonucleotides (Table S2D). Linearization of vector DNA was conducted with the indicated restriction enzyme and plasmids were constructed using Gibson assembly. Sequencing was performed by Eurofins Genomics (Ebersberg, Germany) with the sequencing oligonucleotides listed. (D) Oligonucleotides used in this study. Download Table S2, DOCX file, 0.05 MB. Copyright © 2022 Kever et al. 2022 Kever et al. https://creativecommons.org/licenses/by/4.0/ This content is distributed under the terms of the Creative Commons Attribution 4.0 International license.

The extent of inhibition showed clear differences between the individual phages and aminoglycosides. Remarkably, infection with some phages, namely, the virulent phages Alderaan, Coruscant, and Spe2 as well as the temperate E. coli phage λ, was significantly impaired with increasing aminoglycoside concentrations. In contrast, all phages infecting S. coelicolor, CL31 infecting C. glutamicum MB001, and the T phages, RNA phage MS2, and filamentous phages M13 and fd infecting E. coli displayed no susceptibility to the tested aminoglycosides. The phages susceptible to aminoglycosides infect widely divergent hosts and possess different lifestyles and types of genome ends (Table S2B). However, they are all dsDNA phages belonging to the family *Siphoviridae*, suggesting a specificity of aminoglycosides for this phage family.

**FIG 2:**
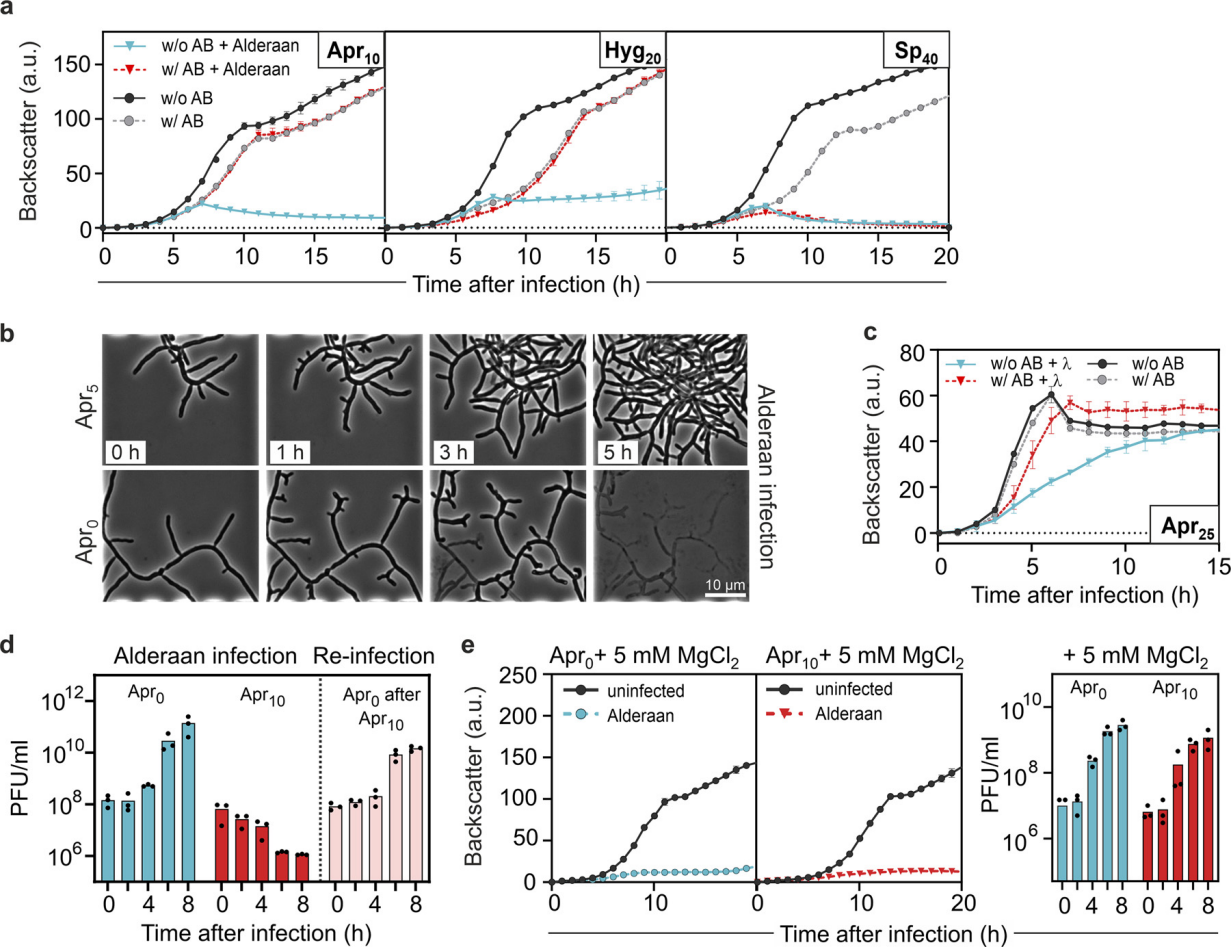
Aminoglycosides strongly inhibit phage amplification in liquid cultures. (a) Infection curves for Streptomyces venezuelae infected by phage Alderaan in the presence of different aminoglycosides (concentrations, in μg/mL, are indicated with subscripts; AB, antibiotic). (b) Time-lapse micrographs of *S. venezuelae* cultivated in a microfluidics system and challenged with Alderaan (insets show time after infection). (c) Infection curves for E. coli DSM 4230 infected by λ in the presence of 25 μg/mL apramycin. (d) Phage titers determined over two successive rounds of infection. A first infection round of *S. venezuelae* by Alderaan was performed in the presence or absence of apramycin. At the end of the cultivation, surviving cells from the apramycin-treated cultures were collected and exposed to phage Alderaan again, this time in the absence of apramycin. (e) Effect of MgCl_2_ on infection of *S. venezuelae* by Alderaan, assessed by infection curves and determination of the corresponding phage titers over time. (a, d, and e) Alderaan was added to an initial titer of 10^7^ PFU/mL; (c) λ was added to an initial titer of 10^8^ PFU/mL. For growth curves and phage titers in panels a, c, d, and e, data are averages for three independent biological replicates (*n* = 3).

In the case of *S. venezuelae* phages, we observed the strongest inhibition with the aminocyclitol antibiotic apramycin. The *S. venezuelae* phage Alderaan showed the highest susceptibility among all tested phages, leading to ~10^6^-fold reduction in numbers of PFU for 25 μg/mL apramycin and a complete inhibition of cell lysis at 100 μg/mL hygromycin or apramycin (Fig. 1b and c). This observation was in line with results from infection assays in liquid culture revealing no more culture collapse when supplementing the respective aminoglycosides (Fig. 2a). The antiviral activity was further demonstrated to be dose dependent, showing already an inhibition of infection at 1 μg/mL apramycin (Fig. S1). In contrast, no antiviral activity was detected for spectinomycin (Fig. 2a).

10.1128/mbio.00783-22.4 FIG S1 Dose-dependent effect of apramycin on the *Streptomyces* phage Alderaan. (a) Growth of Streptomyces venezuelae ATCC 10712 pIJLK04 infected with the phage Alderaan showing the dose-dependent effects of apramycin on infection (*n* = 3 independent biological replicates; error bars represent standard deviations [SD]) (AB; antibiotic). (b) Corresponding phage titers over time in presence of increasing concentrations of apramycin (0, 1, 2.5, and 10 μg/mL). Data are averages for three independent biological replicates. Download FIG S1, PDF file, 1.1 MB. Copyright © 2022 Kever et al. 2022 Kever et al. https://creativecommons.org/licenses/by/4.0/ This content is distributed under the terms of the Creative Commons Attribution 4.0 International license.

To visualize the effect of apramycin on infection dynamics using live-cell imaging, *S. venezuelae* mycelium was grown from spores in a microfluidic device and infected with the phage Alderaan. Addition of apramycin almost completely inhibited phage-mediated lysis of *Streptomyces* mycelium, confirming the protective effect of apramycin against phage infection (Fig. 2b and Video S1).

Infection of E. coli with the model phage λ was also strongly impaired in the presence of aminoglycosides. Here, apramycin and kanamycin at concentrations as low as 25 μg/mL showed a protective effect in liquid cultures (Fig. 2c and Fig. S2a) as well as an up to 1,000-fold reduction in numbers of PFU (Fig. 1b and c). Furthermore, this effect was shown to be independent of the host strain used (Fig. S2b).

10.1128/mbio.00783-22.5 FIG S2 Effect of aminoglycosides on E. coli phage λ. (a) Infection curves of E. coli DSM 4230 infected with phage λ in presence of different aminoglycosides (*n* = 3 independent biological replicates; error bars represent SD). (b) Heat map showing the log_10_ fold change in plaque formation by λ on different E. coli strains in the presence of aminoglycosides relative to the aminoglycoside-free control. (c) Reinfection of cultures previously treated with apramycin (Apr_25_, top row, right), shows efficient infection of E. coli DSM 4230 by phage λ in the absence of apramycin (Apr_25_, bottom row, right). (d) Addition of MgCl_2_ counteracts the effect of apramycin on infection of E. coli DSM 4230 by λ. (e) Potassium efflux assays performed with E. coli DSM 4230 wild type and the E. coli JW3996 *ΔlamB* strain (lacking the λ receptor). λ was added after 5.5 min. Download FIG S2, PDF file, 2.5 MB. Copyright © 2022 Kever et al. 2022 Kever et al. https://creativecommons.org/licenses/by/4.0/ This content is distributed under the terms of the Creative Commons Attribution 4.0 International license.

In the case of temperate phages such as λ, an increased entry into the lysogenic cycle could explain the absence of phage amplification in the presence of aminoglycosides. To test this hypothesis, we conducted a reinfection experiment, in which cells surviving the first round of infection were washed and exposed to the same phage again. In the first infection round, cultures without apramycin showed a strongly increasing phage titer associated with extensive lysis of the culture. In contrast, infection in the presence of apramycin was completely inhibited, showing no phage amplification during λ infection and even an ~100-fold decrease in phage titers over time for Alderaan (Fig. 2d and Fig. S2c).

Interestingly, removal of the antibiotic and reinfection of cells from apramycin-treated cultures resulted in similar amplification kinetics of Alderaan and λ compared to an untreated control. Hence, these results do not support the selection of genetically encoded resistance traits or, in the case of λ, an increased formation of lysogens but rather indicate a reversible antiphage effect of apramycin.

Since elevated Mg^2+^ levels were previously shown to interfere with aminoglycoside uptake (16) and streptomycin-mediated inhibition of phage infection (12), we examined whether the antiviral effect of apramycin is alleviated in the presence of MgCl_2_. As shown in Fig. 2e, phage infection was completely restored by the addition of 5 mM MgCl_2_, as evidenced by the strong growth defect and the increasing phage titer during infection. Comparable results regarding the antagonistic effects of MgCl_2_ were also obtained for λ (Fig. S2d). Overall, these results suggest that the antiviral effect of aminoglycosides is based on an interference with phage infection at the intracellular level, probably during or shortly after phage DNA injection.

### Spent medium of a natural aminoglycoside producer provides protection against phage predation.

**FIG 3:**
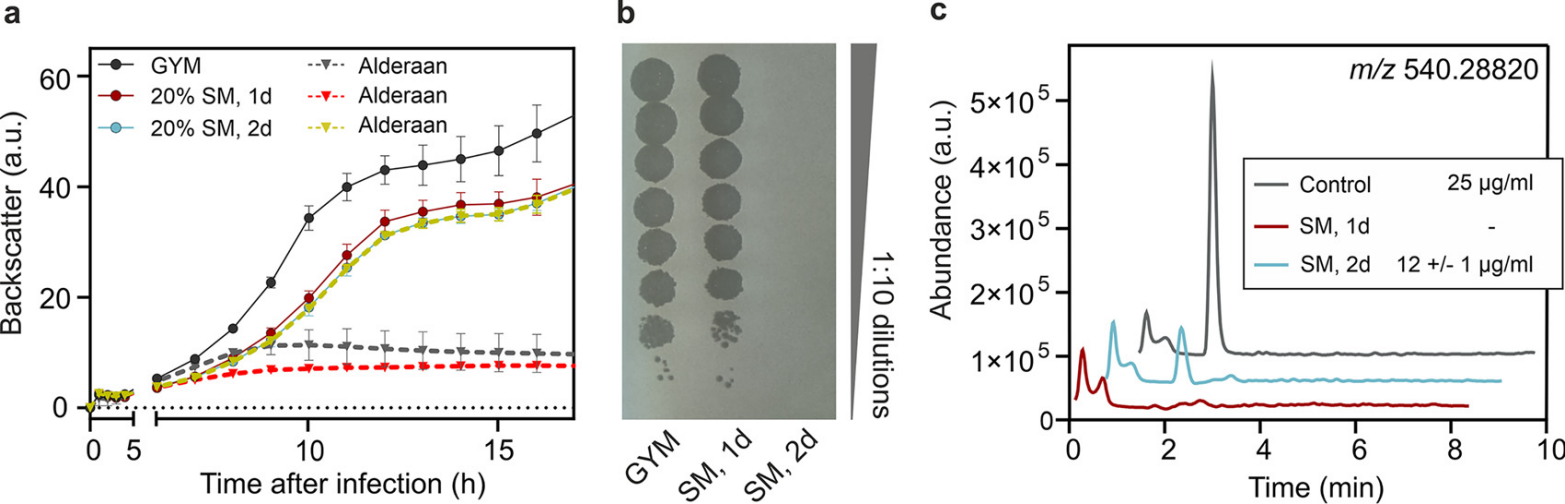
Secondary metabolites produced by Streptoalloteichus tenebrarius inhibit phage infection. (a) Influence of spent medium from *S. tenebrarius* on infection of *S. venezuelae* by Alderaan. Data are averages for three independent biological replicates; error bars represent standard deviations. (b) Determination of the final phage titers of infected cultures shown in panel a. Results are representative of two biological replicates. (c) Extracted ion chromatogram of samples analyzed by LC-MS assessing the presence of apramycin (molecular weight, 539.58 g/mol) in spent medium (SM) of *S. tenebrarius*. The indicated concentrations of apramycin are close to the detection limit under these measuring conditions. GYM, glucose-yeast extract-malt extract medium.

As *Streptomyces* are the natural producers of aminoglycosides, we examined whether infection of *S. venezuelae* in spent medium of the apramycin producer Streptoalloteichus tenebrarius (formerly known as Streptomyces tenebrarius [17]) provides protection against phage predation. Alderaan infection was not impaired by spent medium of *S. tenebrarius* harvested after 1 day of cultivation. In contrast, cultivation in spent medium taken after 2 days completely reproduced the antiviral effect observed during experiments with supplemented purified apramycin, showing equivalent growth of infected and uninfected cultures (Fig. 3a). Endpoint quantification of extracellular phage titers confirmed this inhibition of infection, as no more infective extracellular phages were detectable in the supernatants of the infected cultures (Fig. 3b). Importantly, this protective effect of *S. tenebrarius* spent medium coincided with the presence of apramycin in cultures, as determined by liquid chromatography-mass spectrometry (LC-MS) (Fig. 3c). While the phage-inhibitory effect of the supernatants is very likely to be caused by the native levels of apramycin, we cannot exclude the possibility that this strain may produce other compounds with antiphage properties. Taken together, these data suggest that production of aminoglycoside antibiotics in natural environments might serve as a chemical defense providing protection against phage infection on a community level.

### Aminoglycosides block an early step of phage infection.

**FIG 4:**
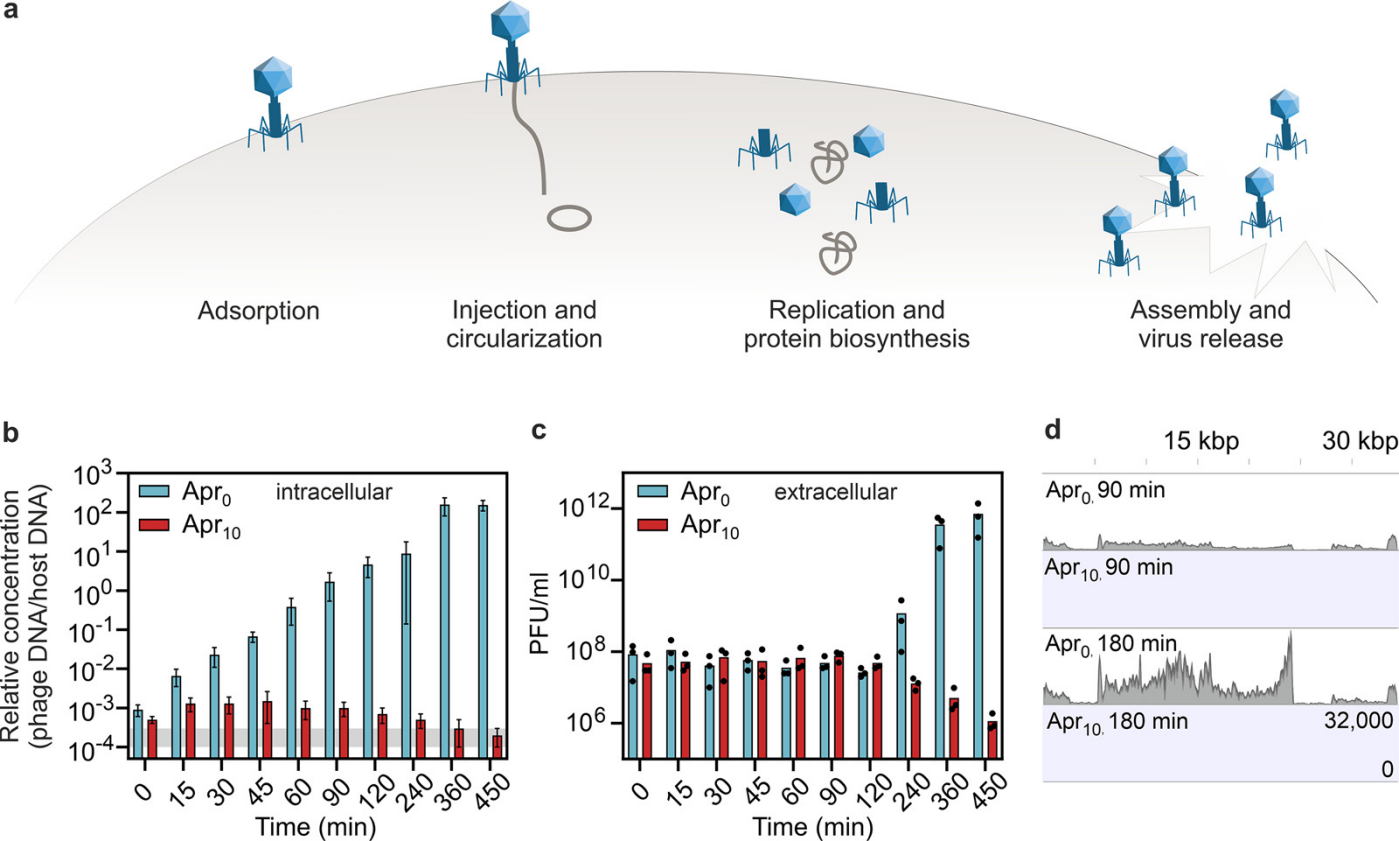
Apramycin blocks the phage life cycle at an early stage—before replication and transcription of phage DNA. (a) Scheme of the phage lytic life cycle, highlighting the different steps which could be inhibited by antiphage metabolites. (b) Infection of *S. venezuelae* by Alderaan; time-resolved quantification of phage DNA by qPCR in the intracellular fraction. To quantify the relative concentration of phage DNA per host DNA, a gene coding for the minor tail protein of Alderaan (HQ601_00028) and the housekeeping gene *atpD* of *S. venezuelae* were used. The corresponding oligonucleotide sequences are provided in Table S2D. Data are means for three independent biological replicates measured as technical duplicates. The range of relative concentrations measured for the uninfected controls (measured 120 min postinfection) is marked in gray. Note that the values measured for apramycin-treated samples are close to or even below the detection limit. (c) Time-resolved determination of Alderaan titers in the extracellular medium via double-agar overlays. *n* = 3 independent replicates. (d) RNA-seq coverage of the Alderaan genome (39 kbp) during infection in the presence and absence of apramycin.

To decipher the mechanism underlying the antiviral activity of aminoglycosides, we investigated the influence of apramycin on the different steps of the phage infection cycle (Fig. 4a).

First, we determined the impact of apramycin on the adsorption step, by following phage titers over time after performing an intense washing 15 min after phage addition to remove Alderaan phages that are only reversibly adsorbed to *Streptomyces* mycelium (Fig. S3). We confirmed that this 15-min preincubation time was sufficient to reach the stage of irreversible adsorption of phage particles, as the control without apramycin showed strongly increasing titers following washing. Importantly, the outcome of phage amplification was determined only in the presence of apramycin in the main culture, as preincubation with apramycin had no influence on later phage titers. Taken together with the adsorption assay performed in the presence of apramycin (Fig. S4a), these data suggest that apramycin does not inhibit irreversible adsorption but rather a later stage of the phage life cycle. In accordance with these findings, preincubation of phage particles with apramycin showed no impact on phage infectivity at physiologically relevant levels of 10 or 50 μg/mL apramycin (Fig. S5). In contrast, higher concentrations (>500 μg/mL) strongly impacted phage infectivity, showing a ~100-fold reduction in PFU/mL after 24 h of incubation.

10.1128/mbio.00783-22.6 FIG S3 Synchronized infection of Streptomyces venezuelae with phage Alderaan under apramycin pressure. Streptomyces venezuelae ATCC 10712 pIJLK04 was inoculated to an OD_450_ of 1 and preincubated with 10^8^ PFU/mL for 15 min at room temperature with gentle shaking. After four washing steps with GYM medium to remove unadsorbed phages, cultures were diluted to a final starting OD_450_ of 0.1. Preincubation with phages and further cultivation was performed with and without apramycin (10 μg/mL) as indicated. (a) Growth of Streptomyces venezuelae infected with phage Alderaan (*n* = 3 independent biological replicates). (b) Corresponding plaque assays showing comparable phage amplification during the main cultivations performed in absence of apramycin, independent of the presence of apramycin in the preincubation step. Download FIG S3, PDF file, 1.1 MB. Copyright © 2022 Kever et al. 2022 Kever et al. https://creativecommons.org/licenses/by/4.0/ This content is distributed under the terms of the Creative Commons Attribution 4.0 International license.

10.1128/mbio.00783-22.7 FIG S4 Investigations of the mechanism of action of apramycin. (a) Effect of apramycin on phage adsorption of phage Alderaan to *S. venezuelae* ATCC 10712 pIJLK04. Shown is the time-resolved quantification of extracellular Alderaan DNA via qPCR using a gene coding for the minor tail protein (HQ601_00028, oligonucleotide sequences are provided in Table S2D). Culture supernatants were pretreated with 100 U/mL DNase to exclusively quantify phage DNA deriving from intact phage particles. A DNase-treated phage stock with known phage titer was used to infer phage titers (in PFU/mL) from DNA quantification. Data are means for two independent biological replicates measured as technical duplicates. (b) Impact of apramycin (10 μg/mL) when added at the different indicated time points after phage infection. For each sample, phage titers were measured over time. Data are averages for two independent biological replicates. (c) Enlargement of Fig. 4c showing the RNA-seq coverage of the Alderaan genome in presence or absence of apramycin. Genome organization of Alderaan is displayed at the top. Download FIG S4, PDF file, 1 MB. Copyright © 2022 Kever et al. 2022 Kever et al. https://creativecommons.org/licenses/by/4.0/ This content is distributed under the terms of the Creative Commons Attribution 4.0 International license.

10.1128/mbio.00783-22.8 FIG S5 Preincubation of phage Alderaan with apramycin. Alderaan phages were preincubated in GYM medium containing the indicated apramycin concentrations at 30°C and 900 rpm before spotting on a bacterial lawn of Streptomyces venezuelae ATCC 10712 pIJLK04. Download FIG S5, PDF file, 1.1 MB. Copyright © 2022 Kever et al. 2022 Kever et al. https://creativecommons.org/licenses/by/4.0/ This content is distributed under the terms of the Creative Commons Attribution 4.0 International license.

Next, we assessed phage DNA delivery and amplification by determining the level of intracellular Alderaan DNA during infection via quantitative real-time PCR (qPCR). In the absence of apramycin, the phage DNA levels increased exponentially until 360 min postinfection, indicating active genome replication across several rounds of infection (Fig. 4b). Simultaneous measurement of extracellular phage titers showed stable titers until 120 min, followed by a strong rise indicative of the release of new phage progeny after cells lysis (Fig. 4c). Conversely, only a slight increase in intracellular DNA was obtained for infection under apramycin pressure (Fig. 4b; note that measurement in the presence of apramycin is close to the detection limit). Relative phage concentrations then declined starting at 45 min and were even similar to those measured in the uninfected controls at 360 and 450 min, hinting at degradation of intracellular phage DNA. In the meantime, extracellular phage titers of apramycin-treated cultures declined from 120 min (Fig. 4c). Overall, these results suggest an inhibition of phage genome replication but do not exclude an interference with the injection process in *S. venezuelae*.

Assuming that apramycin blocks an early step of phage infection prior to genome replication, addition of the antibiotic after the replication phase would not interfere with the infection. This hypothesis was indeed confirmed by supplementation of the aminoglycoside at different time points post infection (Fig. S4b). Corresponding infection assays indicated that apramycin addition 30 min after infection was sufficient to prevent a reproductive Alderaan infection. The observed decrease in extracellular phage titers is probably the result of adsorption and subsequent DNA injection of a fraction of phages without release of new infective viral particles.

In contrast, no decrease in extracellular phage titers was observed when apramycin was added 1 to 2 h after infection, indicating that the first phages were able to complete their infection cycle before apramycin was added. Comparison of these results with the quantification of intracellular phage DNA (Fig. 4b) further showed that this period corresponds to the replication phase, indicating that replication is a sensitive time point for the antiviral activity of aminoglycosides. In the case of the E. coli system, the measurement of potassium efflux is an established approach to probe the successful delivery of phage DNA into the bacterial cell (18). Applying this method to infection of E. coli with phage λ confirmed that the injection process was not impaired by apramycin (Fig. S2e).

Next, we examined the influence of apramycin on phage DNA transcription. RNA sequencing revealed an increasing transcription of Alderaan DNA during phage infection under normal infection conditions, whereas addition of apramycin drastically hindered phage gene expression (Fig. 4d and Fig. S4c). In accordance with the previous results, these data suggest a blockage of phage infection prior to phage DNA replication and transcription, which is congruent with a recent report of inhibition of two mycobacteriophages by streptomycin, kanamycin, and hygromycin (19).

**FIG 5:**
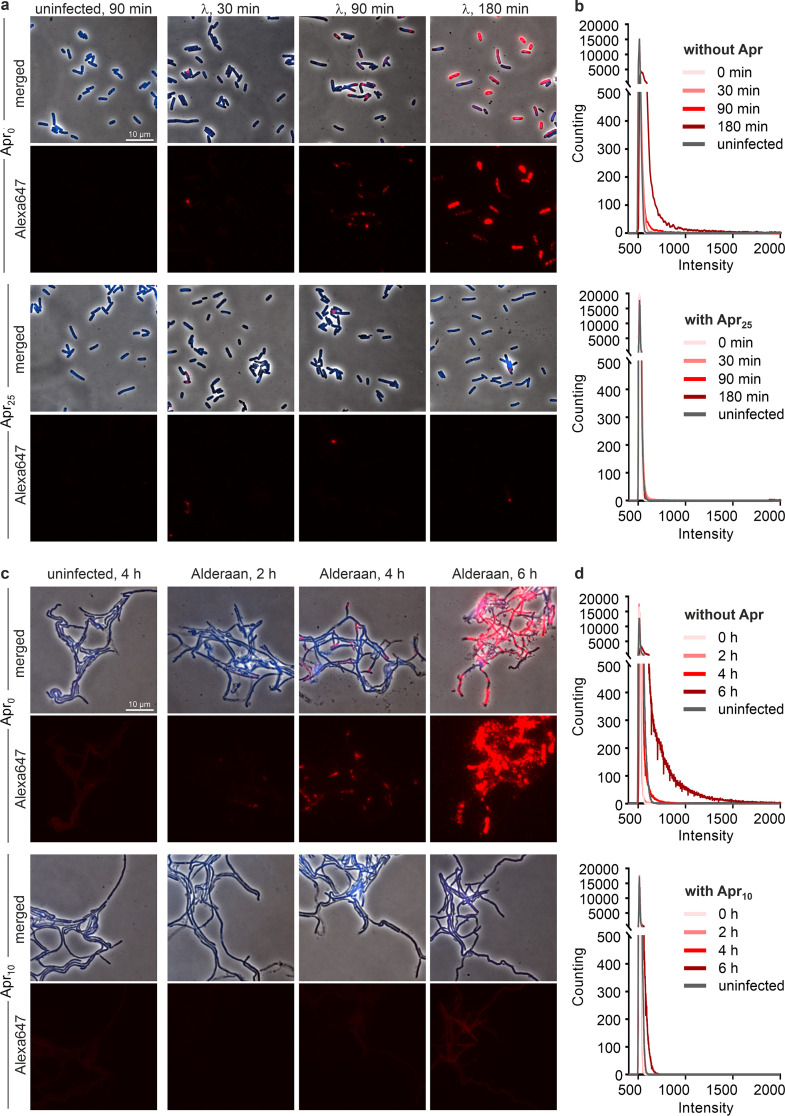
Visualization of intracellular phage DNA by phage targeting direct-geneFISH. (a and c) Phage-targeting direct-geneFISH micrographs of (a) E. coli DSM4230 infected with λ and (c) *S. venezuelae* infected with Alderaan in the presence and absence of 25 μg/mL and 10 μg/mL apramycin, respectively. (First and third rows) Phase-contrast pictures merged with fluorescence signal from bacterial DNA (DAPI, blue) and phage DNA (Alexa647, red). (Second and fourth rows) Fluorescence signal from phage DNA only (Alexa647, red). Bar, 10 μm. (b and d) Quantification of Alexa647 fluorescence in (b) E. coli cells infected with λ and (d) *S. venezuelae* cells infected with Alderaan, shown as density plots of pixel counts relative to their fluorescence intensity. Data are averages for biological three independent biological replicates (*n* = 3); the data for all replicates are shown in Fig. S6a and b.

To visualize intracellular phage infections in the presence and absence of apramycin, we performed fluorescence *in situ* hybridization of phage DNA (phage-targeting direct-geneFISH) using Alexa Fluor 647-labeled probes specific for the phage genome. In this assay, the formation of bright and distinct fluorescent foci is indicative of advanced viral infections (20). When infecting E. coli with λ, comparable amounts of injected phage DNA were detected for both infection conditions after 30 min. This result is in line with the potassium efflux assay described above, which showed similar injection kinetics in the presence of apramycin for E. coli (Fig. S2e). As the infection progressed, only samples without apramycin exhibited a strong increase in fluorescence intensity 90 min and 180 min postinfection, further hinting at an inhibited replication in the presence of apramycin (Fig. 5a). For Alderaan, an increase in red fluorescence and thus intracellular phage DNA could be observed 4 h after infection and was even more pronounced at 6 h, reflecting phage DNA replication. In contrast, apramycin-treated samples showed only a very weak and more diffuse red fluorescent signal in the 6-h samples (Fig. 5c), which is overall consistent with the quantification of intracellular phage DNA by qPCR (Fig. 4b). Plotting the distribution of fluorescence intensity per pixel confirmed that the massive increase in fluorescence at the last time point (180 min for λ and 6 h for Alderaan, respectively) was inhibited in the presence of apramycin, supporting the blockage of replication exerted by apramycin (Fig. 5b and d; Fig. S6a and c). Interestingly, determination of the percentage of λ-infected E. coli cells over time showed a peak at 30 min in apramycin-treated samples followed by a decline down to almost no infected cell at 180 min (Fig. S6b). This observation suggests that intracellular phage DNA was degraded following the halt of the phage life cycle caused by apramycin.

10.1128/mbio.00783-22.9 FIG S6 Distribution of fluorescence intensities from phage targeting direct-geneFISH. (a and c) Quantification of Alexa647 fluorescence in (a) E. coli cells infected with λ and (c) *S. venezuelae* cells infected with Alderaan, shown as density plots of pixel counts relative to their fluorescence intensity. For each panel, profiles of the three biological replicates are shown. (b) Determination of the percentage of E. coli cells infected with λ over time (*n* = 3 independent biological replicates) (Uninf, uninfected). A cell was considered infected if Alexa647 (red) fluorescence was detected within the cell. Download FIG S6, PDF file, 1.1 MB. Copyright © 2022 Kever et al. 2022 Kever et al. https://creativecommons.org/licenses/by/4.0/ This content is distributed under the terms of the Creative Commons Attribution 4.0 International license.

### Acetylation of apramycin abolishes its antibacterial, but not antiphage properties.

Enzymatic modification of aminoglycosides is a major mechanism of bacterial resistance to these antibiotics. Aminoglycoside-modifying enzymes are categorized in three major classes: aminoglycoside *N*-acetyltransferases (AACs), aminoglycoside *O*-nucleotidyltransferases (ANTs), and aminoglycoside *O*-phosphotransferases (APHs) (13). Addition of an acetyl, adenyl, or phosphoryl group at various positions of the aminoglycoside core scaffold decreases the binding affinity of the drug for its primary ribosomal target, leading to the loss of the antibacterial potency, with the modified aminoglycosides being described as “inactivated.”

**FIG 6:**
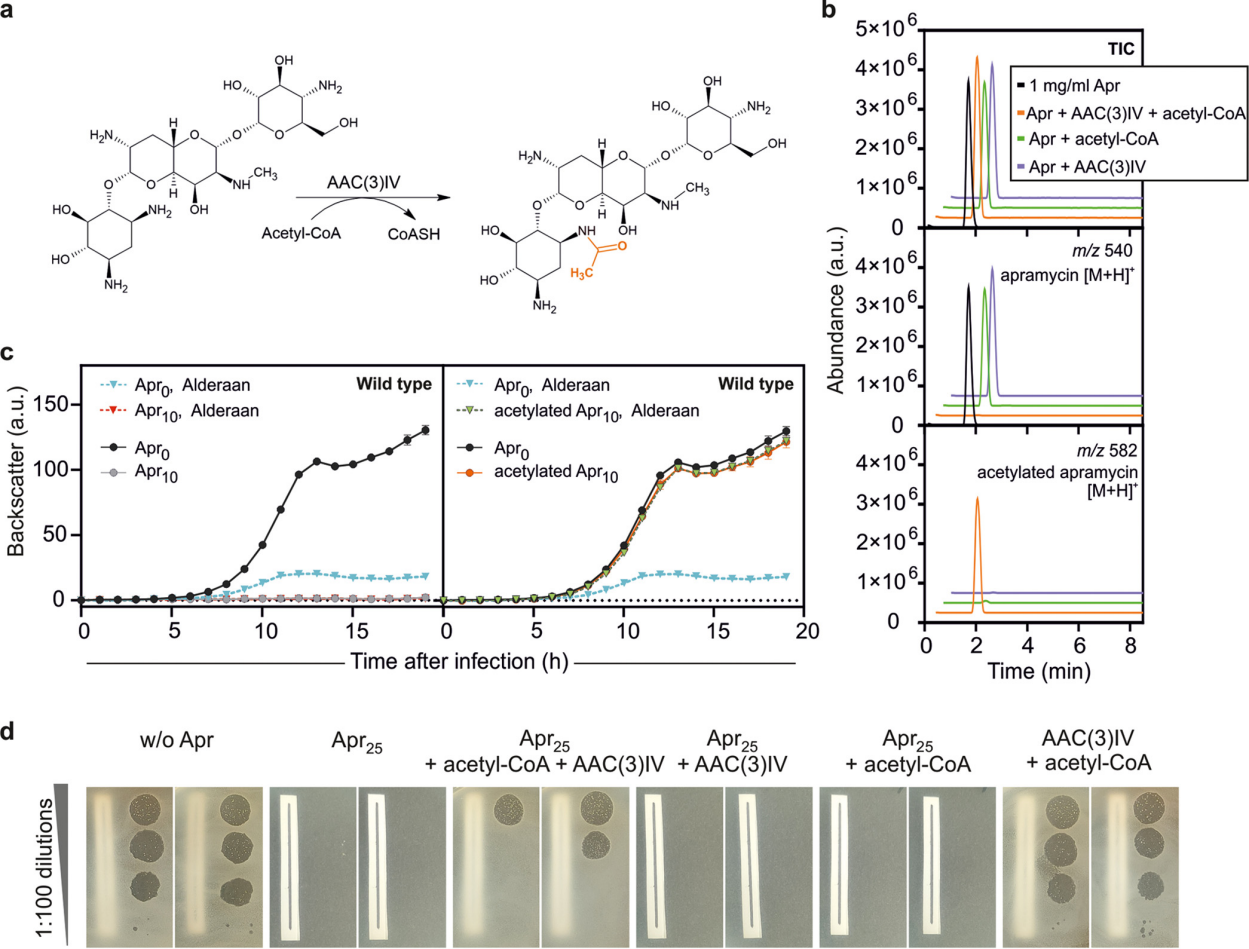
Acetylated apramycin strongly inhibits phage infection, despite the loss of its antibacterial properties. (a) Acetylation reaction of apramycin catalyzed by the AAC(3)IV acetyltransferase. (b) Total ion chromatogram and extracted ion chromatograms of samples analyzed by LC-MS assessing the presence of apramycin (molecular weight, 539.58 g/mol; *m/z* 540) and acetylated apramycin (molecular weight, 581.62 g/mol; *m/z* 582) after *in vitro* acetylation of apramycin. (c and d) Effect of acetylated apramycin on infection of wild-type *S. venezuelae* with Alderaan, performed in liquid (c) and solid (d) media. For panel d, the reaction mixtures of the *in vitro* acetylation assays containing apramycin, acetyl-CoA, the AAC(3)IV acetyltransferase, or different combinations of these were used to supplement the plates. A piece of paper was placed below plates to facilitate assessment of bacterial growth.

However, the impact of these modifications on the antiphage activity of aminoglycosides is unknown. We set out to answer this question using apramycin and the acetyltransferase AAC(3)IV (21), also referred to as “Apr” in the literature. In the presence of apramycin, AAC(3)IV catalyzes the acetylation of the 3-amino group of the deoxystreptamine ring, using acetyl coenzyme A (acetyl-CoA) as a cosubstrate (Fig. 6a).

Using purified AAC(3)IV enzyme, we performed an *in vitro* acetylation reaction of apramycin. LC-MS analysis of the reaction mixtures revealed complete acetylation of apramycin, as the peak of apramycin (*m/z* 540) disappeared in favor of the one corresponding to acetylated apramycin (*m/z* 582) (Fig. 6b).

The efficiency of the acetylation reaction being confirmed, we tested the effect of acetylated apramycin on phage infection in liquid medium, using wild-type *S. venezuelae* (not carrying a plasmid-borne acetyltransferase gene) and its phage Alderaan. As expected, apramycin fully prevented growth of *S. venezuelae*, while acetylated apramycin did not show any toxicity effect. Strikingly, phage infection was completely inhibited in the presence of acetylated apramycin, suggesting that acetylation of apramycin does not interfere with its antiphage properties (Fig. 6c). Plate assays showed a comparable pattern: acetylation of apramycin suppressed its antibacterial effect but did not disrupt its ability to inhibit phage infection (Fig. 6d). Altogether, these results suggest a decoupling of the antibacterial and antiphage properties of apramycin and further highlight the distinct molecular target accounting for apramycin’s antiphage properties.

## DISCUSSION

We have shown that aminoglycosides inhibit phage infection in a diverse set of bacterial hosts by blocking an early step of the phage life cycle prior to DNA replication. These findings highlight the multifunctionality of this class of antibiotics, as they possess both antibacterial and antiviral properties. The dual properties of aminoglycosides were first recognized in the 1950s and 1960s (10–12, 22), but mechanistic studies about their impact on phage infection differed in their conclusions. Brock and colleagues proposed a 2-fold inhibition of streptomycin on Enterococcus faecium, where streptomycin would be able to inhibit both genome injection and replication (12). In the same year, it was proposed that streptomycin inhibits the process of injection of these phages by preventing proper unfolding of the phage genome through cross-linking of the phage DNA (23). Recently, Jiang and colleagues reported the inhibition of two M. tuberculosis phages by streptomycin, kanamycin, and hygromycin (19). Following adsorption and quantifying of viral DNA, the authors proposed that the blockage caused by aminoglycosides occurs between genome circularization and replication. Our results put forward different pictures depending on the bacterial host. Infection with λ and Alderaan phages seems to be blocked at the genome replication stage by apramycin in both cases. However, we cannot exclude some additional interference with the injection step of phage Alderaan. This disparity presumably has its roots in the major differences in cell wall architectures between Gram-positive and -negative bacteria. Moreover, it opens the possibility that aminoglycosides exert a multilayered inhibition of phage infection in their natural producers.

More recently, sublethal aminoglycoside concentrations of aminoglycosides were shown to inhibit phage infection in E. coli and Bacillus cereus (24). Interestingly, tetracycline, another translation-inhibiting antibiotic binding to the 30S ribosome, was much less effective at suppressing phage proliferation. This difference suggests a direct antiphage action of aminoglycosides and indicates that inhibition of phage replication is not a common trait of antibiotics blocking protein synthesis.

One crucial question is that of which structural features or chemical groups of aminoglycosides are responsible for their antiphage properties. Our screening revealed that aminoglycosides belonging to 3 of 4 subclasses showed antiphage activity, suggesting that these properties are widespread among aminoglycosides and not limited to one particular subclass. Furthermore, a potential antiviral activity is probably also strongly influenced by the uptake and cell envelope structure of a particular host species. However, thorough structure-function relationship studies are needed to address this topic.

The versatility of aminoglycosides can be attributed to their ability to bind a wide variety of molecules, including nucleic acids—DNA or RNA, biologically or nonbiologically derived. The most prominent target of aminoglycosides is the 16S rRNA, accounting for the disruption of protein translation and hence their bactericidal properties (13). Aminoglycosides have also been shown to bind to seemingly unrelated families of RNA molecules such as group I introns (25), a hammerhead ribozyme (26), the *trans*-activating response element (TAR) (27) and the Rev response element (RRE) of the human immunodeficiency virus (HIV) (28–30). Interestingly, this effect on HIV is the only report of a direct inhibition of eukaryotic viruses by aminoglycosides. Evidence of indirect influence on infection by eukaryotic viruses comprises the activation of interferon-based antiviral response following topical application of aminoglycosides (31), and the enhancement of plaque formation by coxsackieviruses via increased diffusion of virions in the extracellular matrix (32). Furthermore, *in vitro* studies showed condensation of purified phage λ DNA by aminoglycosides. It was proposed that the clamp formed by aminoglycosides around the DNA double helix causes a bend responsible for the formation of toroids and other structural deformations (33, 34).

Injected phage DNA is linear, in a relaxed state, and not protected by DNA-binding proteins, and it is therefore probably highly sensitive to DNA-binding molecules. Interestingly, anthracyclines—another class of secondary metabolites produced by *Streptomyces* strains with antiphage properties—inhibit phage infection at a similar stage (5). While the exact mechanism of action underlying phage inhibition by anthracyclines and aminoglycosides remains elusive, these recent results suggest that already injected but not yet replicating phage DNA is preferentially targeted by antiviral molecules. Repeated efforts to isolate Alderaan clones that developed resistance to apramycin were not successful, suggesting that phage inhibition by apramycin relies on structural properties of phage DNA that cannot be readily overcome by single-base mutations or small structural variants.

Therapeutical use of phages—known as phage therapy—is often combined with an antibiotic treatment due to the potentially synergistic effect between these two antimicrobial agents. In contrast, we describe here an antagonistic impact of a common antibiotic class on phages, which has important implications for phage-aminoglycoside combination treatment. We propose that sensitivity of the phage to aminoglycosides be assessed *in vitro* before administration of such combination therapy.

From a more fundamental perspective, these findings also shed new light on the role of aminoglycosides in natural bacterial communities. While their use as antibiotics for medical applications has been extensively documented, until now, relatively little was known about their function in the natural setting. We posit that aminoglycosides not only are used by their producers as a powerful weapon against bacterial competitors but also protect them against phage predation at the community level. In streptomycetes, antibiotic production happens mainly at later stages of development, typically during the formation of aerial hyphae (35–37), while phages preferentially attack young mycelium (38). This clear difference in chronology may make secondary-metabolite-mediated antiphage defense seem irrelevant when studied in a laboratory setting. However, this defense strategy takes its full meaning in the light of community ecology, where older fractions of an established microbial community could ensure a protective “antiviral milieu” for their descendants.

Another key consideration to appreciate aminoglycoside antiviral properties in an ecological context concerns the importance of the resistance mechanism to these antibiotics. Using Streptomyces venezuelae and its phage Alderaan, we showed that acetylation of apramycin led to a loss of its antibacterial properties, while leaving its ability to block phage infection untouched. Assuming that this observation can be extended to more phages and aminoglycoside-modifying enzymes, it raises the question of whether deflecting the antibacterial effect of aminoglycosides while benefiting from their intracellular protective effect against phages would be a strategy favored over antibiotic resistance by efflux. Interestingly, unlike many antibiotic classes (39), efflux proteins reported to pump out aminoglycosides are relatively rare and conferred only partial resistance to aminoglycosides (13). In contrast, aminoglycoside-modifying enzymes are widespread and found in natural producers and clinical isolates alike (13, 40). Natural aminoglycoside producers often encode a second line of resistance represented by 16S rRNA methyltransferases, whose action makes their ribosomes insensitive to aminoglycosides without interfering with the action of the latter on phages (40).

Considering the colossal number of molecules produced by environmental bacteria whose physiological role is still unclear, we postulate that additional prokaryotic antiphage metabolites are to be discovered in the future, further underlining the extraordinary diversity of strategies employed by bacteria against their viral predators.

## MATERIALS AND METHODS

### Bacterial strains and growth conditions.

All bacterial strains, phages, and plasmids used in this study are listed in Table S2A, B, and C, respectively. For growth studies and double-agar overlay assays, *Streptomyces* sp. cultures were inoculated from spore stocks and cultivated at 30°C and 120 rpm using glucose-yeast extract-malt extract (GYM) medium for *S. venezuelae* and Streptoalloteichus tenebrarius and yeast extract-malt extract (YEME) medium for S. coelicolor (35). E. coli was cultivated in lysogeny broth (LB) medium at 37°C and 170 rpm, while C. glutamicum was grown in brain heart infusion (BHI) medium at 30°C and 120 rpm.

For double-agar overlays, BHI agar for C. glutamicum, LB agar for E. coli, and GYM agar (pH 7.3) for all *Streptomyces* species were used, with 0.4% and 1.5% agar for the top and bottom layers, respectively. For quantification of extracellular phages, 2 μL of the culture supernatants was spotted on a bacterial lawn propagated on a double-agar overlay inoculated at an initial optical density at 450 nm (OD_450_) of 0.4 for *Streptomyces* spp., an OD_600_ of 0.1 for E. coli, and an OD_600_ of 0.7 for C. glutamicum. Both agar layers were supplemented with antibiotics at the indicated concentrations.

For standard cloning applications, E. coli DH5α was cultivated in LB medium containing the appropriate antibiotic at 37°C and 120 rpm. For conjugation between *Streptomyces* spp. and E. coli, the conjugative E. coli strain ET12567/pUZ8002 was used (41).

### Recombinant DNA work and cloning.

All plasmids and oligonucleotides used in this study are listed in Table S2C and D, respectively. Standard cloning techniques such as PCR and restriction digestion were performed according to standard protocols (42). In all cases, Gibson assembly was used for plasmid construction (43). DNA regions of interest were amplified via PCR using the indicated plasmid DNA as the template. The plasmid backbone was cut using the listed restriction enzymes. DNA sequencing and synthesis of oligonucleotides was performed by Eurofins Genomics (Ebersberg, Germany).

### Phage infection curves.

For phage infection curves, the BioLector microcultivation system of m2p-labs (Baesweiler, Germany) was used (44). Cultivations were performed as biological triplicates in FlowerPlates (m2p-labs, Germany) at 30°C and a shaking frequency of 1,200 rpm. During cultivation, biomass was measured as a function of backscattered light intensity with an excitation wavelength (λ_Ex_) of 620 nm (filter module: λ_Ex_/λ_Em_, 620 nm/620 nm; gain, 25 or 20 in Fig. 3a) every 15 min. All growth curves are baseline corrected. Main cultures of *Streptomyces* spp. in 1 mL GYM medium containing the indicated supplements were inoculated with overnight cultures in the same medium to an initial OD_450_ of 0.15. Infection was performed by adding phages to an initial titer of 10^7^ PFU/mL. Supernatants were collected in 2-h intervals to determine the time course of phage titer via double-agar overlays. Phage infection curves in E. coli were done in the same way at 37°C and 1,200 rpm using an initial OD_600_ of 0.1 in 1 mL LB medium and an initial phage titer of 10^8^ PFU/mL, resulting in a multiplicity of infection (MOI) of 1.

Phage infection curves in shaking flasks were performed analogously to the cultivation in microbioreactors using a shaking frequency of 120 rpm. To study phage infection and the influence of aminoglycosides in *Streptomyces*, we draw attention to the importance of ion content, e.g., of water used for medium preparation.

### Cultivation and perfusion in microfluidic devices.

Single-cell analysis of *S. venezuelae* cells infected with phage Alderaan in presence and absence of apramycin was performed using an in-house-developed microfluidic platform (45–47). Cultivation and time-lapse imaging were performed in three steps. First, cultivation chambers in the microfluidic chip were inoculated with GYM medium containing an initial spore titer of 10^8^ PFU/mL. During the following precultivation phase, cells in all chambers were cultivated under continuous GYM medium supply supplemented with 2.5 μg/mL apramycin (flow rate, 300 nL/min) to allow comparable growth conditions. After 6 h of precultivation, cells were cultivated for 3 h in GYM medium containing one of the final apramycin concentrations (0, 5, or 10 μg/mL). Subsequently, infection was initiated by a continuous supply of GYM medium containing the final apramycin concentrations and Alderaan phages with a titer of 10^8^ PFU/mL (flow rate, 200 nL/min). By using disposable syringes (Omnifix-F tuberculin, 1 mL; B. Braun Melsungen AG, Melsungen, Germany) and a high-precision syringe pump system (neMESYS; Cetoni GmbH, Korbussen, Germany), continuous medium supply and waste removal were achieved. Phase-contrast images were obtained at 5-min intervals (exposure time, 100 ms) by a fully motorized inverted Nikon Eclipse Ti microscope (Nikon Europe B.V., Amsterdam, Netherlands). During the complete cultivation, the temperature was set to 30°C using an incubator system (PeCon GmbH, Erbach, Germany).

### Cultivation in spent medium.

For preparation of spent medium, cultures of the natural apramycin producer Streptoalloteichus tenebrarius were prepared by inoculating 50 mL of GYM medium to an initial OD_450_ of 0.1 and were cultivated for 4 days. Spent medium of the culture was collected every day by centrifugation and subsequent filtration of the supernatant. After adjustment of the pH to 7.3, GYM medium and spent medium were mixed in a ratio of 4:1, so that spent medium accounted for 20% of the total volume. Ten-times-concentrated GYM was added to keep the concentration of C sources equal to that of fresh GYM medium. Cultivation and infection of the apramycin-resistant *S. venezuelae*/pIJLK04 strain in 20% spent medium was conducted in microbioreactors as describe above by using an initial OD_450_ of 0.5 and an initial phage titer of 10^6^ PFU/mL.

### LC-MS measurements of apramycin.

Aminoglycosides were analyzed using an Agilent ultrahigh-performance LC (UHPLC) 1290 Infinity system coupled to a 6130 Quadrupole LC-MS system (Agilent Technologies, Waldbronn, Germany). LC separation was carried out using an InfinityLab Poroshell 120 2.7-μm EC-C_18_ column (3.0 by 150 mm; Agilent Technologies, Waldbronn, Germany) at 40°C. For elution, 0.1% acetic acid (solvent A) and acetonitrile supplemented with 0.1% acetic acid (solvent B) were applied as the mobile phases at a flow rate of 0.3 mL/min. A gradient elution was used, where the amount of solvent B was increased stepwise: minutes 0 to 6, 10% to 25%; minutes 6 to 7, 25% to 50%; minutes 7 to 8, 50% to 100%; and minutes 8 to 8.5, 100% to 10%. The mass spectrometer was operated in the positive electrospray ionization (ESI) mode, and data were acquired using the selected-ion-monitoring (SIM) mode. An authentic apramycin standard was obtained from Sigma-Aldrich (Munich, Germany). Area values for [M+H]^+^ mass signals were linear for metabolite concentrations from 10 to 50 μg/mL.

### Potassium efflux assays.

Cultures of E. coli DSM 4230/pEKEx2.d were grown in LB medium supplemented with 50 μg/mL apramycin at 37°C and 170 rpm overnight. Fresh LB medium (50 μg/mL apramycin if needed) was inoculated 1:100 from the overnight cultures and incubated at 37°C and 120 rpm for 1.5 h. The cultures were centrifuged at 5,000 × *g* for 20 min, and the pellets were resuspended in SM buffer (0.1 M NaCl, 8 mM MgSO_4_, 50 mM Tris-HCl [pH 7.5]). The OD_600_ was measured and adjusted to 2. The cultures were stored at 4°C and incubated at 37°C for 5 min directly before use. The measurements were performed using an Orion potassium ion selective electrode (Thermo Fisher Scientific, Waltham, MA, USA). Five microliters of the prepared cultures was mixed 1:50 with Orion ionic strength adjuster (ISA) (Thermo Fisher Scientific, Waltham, MA, USA), and measurements were started immediately to monitor the electric potential (in millivolts) every 5 s for a total of 60 min at room temperature with constant stirring. If apramycin was needed, it was added in the beginning to a concentration of 100 μg/mL. After 5.5 min, 100 μL of a polyethylene glycol (PEG)-precipitated λ phage lysate in SM buffer (10^11^ PFU/mL) was added to the cultures.

### Quantitative real-time PCR.

Quantification of cell-associated Alderaan phages was performed via quantitative real-time PCR. For this, infection of the apramycin-resistant strain *S. venezuelae* ATCC 10712 pIJLK04 with Alderaan was performed as described in “Phage infection curves.” At the indicated time points, 3 OD units of cells were harvested via centrifugation at 5,000 × *g* and 4°C for 10 min and washed twice with phosphate-buffered saline (PBS) before being stored at −20°C. For quantification of intracellular phage DNA in presence and absence of apramycin, cells were resuspended in 500 μL lysis buffer (10 mM Tris, 50 mM NaCl [pH 7.0]), and cell disruption was performed using a Precellys instrument (Bertin, Montigny Le Bretonneux, France) at 6,000 rpm three times for 40 s each. After centrifugation at 16,000 × *g* and 4°C for 10 min, DNA concentrations in the supernatants were determined via nanophotometer (Implen, Munich, Germany) and adjusted to 1 ng/μL. Finally, 5 μL of the diluted supernatants as the template DNA was mixed with 10 μL 2× Luna universal qPCR master mix (New England BioLabs, Ipswich, MA, USA) and 1 μL of each oligonucleotide (Table S2D) (final oligonucleotide concentration, 0.5 μM) and adjusted to a final volume of 20 μL with double-distilled water (ddH_2_O). Measurements were performed in 96-well plates in the qTOWER 2.2 (Analytik Jena, Jena, Germany). For the determination of the relative concentration of cell-associated phages, the relative expression ratio of the phage target phage gene (HQ601_00028, coding for the minor tail protein of Alderaan; PCR product, 144 bp) to the *S. venezuelae* housekeeping gene *atpD* (coding for the ATP synthase beta subunit; PCR product, 147 bp) was calculated via the “Relative quantification method” function of the qPCRsoft 3.1 software (Analytik Jena, Jena, Germany).

### Transcriptomics via RNA sequencing.

To compare transcription of phage and host DNA in presence and absence of apramycin, infection of the apramycin-resistant strain *S. venezuelae* ATCC 10712/pIJLK04 with Alderaan was conducted as described in “Phage infection curves.” Cells were harvested 90 min and 180 min after infection on ice at 5,000 × *g* and 4°C for 10 min. RNA purification was done using the Monarch total RNA miniprep kit (New England Biolabs, Ipswich, MA, USA) according to the manufacturer's manual. Depletion of rRNA, library preparation, and sequencing were conducted by Genewiz (Leipzig, Germany).

After sequencing, all subsequent steps were conducted using CLC genomic workbench V. 20.0.4 software (Qiagen, Hilden, Germany). The initial quality check to analyze read quality and sequencing performances was followed by a trimming step. This step was used to remove read-through adapter sequences, leftover adapter sequences, low-quality reads (limit = 0.05), and ambiguous nucleotides. Subsequently, the trimmed reads were mapped against the genomes of *S. venezuelae* (accession no. NC_018750.1) and the phage Alderaan (accession no. MT711975.1). Coverage plots were generated to show the distribution of mapped reads on both genomes. Subsequently, transcripts-per-million (TPM) values were calculated using the RNA-seq analysis tool of CLC genomics workbench (read alignment parameters: mismatch cost, 2; insertion cost, 3; deletion cost, 3; length fraction, 0.8; similarity fraction, 0.8; strand specificity, both; maximum number of hits for a read, 10). A table containing these values and an overview matrix containing all values were exported for each sample.

### Phage targeting direct-geneFISH.

Visualization and quantification of intracellular phage DNA during the time course of infection were conducted via fluorescence *in situ* hybridization (FISH), following the direct-geneFISH protocol (48), with modifications as described below.

Design of phage gene probes was done using the gene-PROBER (49). Sequences of the 200-bp polynucleotides for Alderaan and 300-bp polynucleotides for λ are provided in Table S3. Phage infection was performed as described in “Phage infection curves” using 10^7^ PFU/mL as the initial phage titer for both phages. For infection of E. coli, the chemical labeling of polynucleotides with Alexa Fluor 647 dye (Thermo Fisher Scientific, Waltham, MA, USA) as well as the “core” direct-geneFISH protocol for microscopic slides was conducted as described previously using 0.5 mg/mL lysozyme for permeabilization and 35% (vol/vol) formamide during the hybridization step. Imaging of cells was performed with an inverted time-lapse live cell microscope (Nikon Europe B.V., Amsterdam, Netherlands) using a 100× oil immersion objective (CFI Plan Apo Lambda DM; 100× oil; numerical aperture [NA], 1.45; Nikon Europe B.V., Amsterdam, Netherlands) (45). Fluorescence was recorded using the optical filters DAPI (4′,6-diamidino-2-phenylindole) and CY5-4040C (DAPI: excitation, 360/40 nm; dichroic, 400 nm; emission, 460/50 nm; exposure time, 500 ms; CY5: excitation, 628/40 nm; dichroic, 660 nm; emission, 692/40 nm; exposure time, 500 ms [AHF Analysentechnik AG, Tübingen, Germany]). Phase contrast was imaged with an exposure time of 500 ms.

10.1128/mbio.00783-22.3 TABLE S3 Polynucleotides used for phage targeting direct-geneFISH. Download Table S3, DOCX file, 0.02 MB. Copyright © 2022 Kever et al. 2022 Kever et al. https://creativecommons.org/licenses/by/4.0/ This content is distributed under the terms of the Creative Commons Attribution 4.0 International license.

For *S. venezuelae* infection, the protocol was adjusted as follows. Fixation of cells and phages was performed in 50% ethanol overnight at 4°C. After washing and immobilization, permeabilization was performed with 1.5 mg/mL lysozyme for 60 min at 37°C. Due to the high GC content of the phage Alderaan, the formamide concentration in the hybridization buffer and in the humidity chamber was adjusted to 60% (vol/vol) and the NaCl concentration in the washing buffer was reduced to 4 mM. After counterstaining with DAPI, imaging of cells was performed as described for E. coli using the optical filters DAPI and CY5-4040C with the indicated exposure times (DAPI: excitation, 360/40 nm; dichroic, 400; emission, 460/50 nm; exposure time, 800 ms; CY5: excitation, 628/40 nm; dichroic, 660; emission, 692/40 nm; exposure time, 500 ms [AHF Analysentechnik AG, Tübingen, Germany]). Phase contrast was imaged with an exposure time of 500 ms. The images for phage signal quantification were taken at the same exposure times to enable comparison; exposure times were adjusted to avoid overexposure of the signals. Preparation of image cutouts and adjustments of lookup tables (LUTs) were performed using NIS-Elements BR 5.30.03 (64 bit).

As a quantification of the microscopic analyses, plots showing the distribution of Cy5 signal intensities for single microscopy images were generated. To this end, signal intensity of each pixel of the Cy5 channel images was determined using the software Fiji (50), and the frequency of occurrence of each intensity was calculated and plotted using R with the Rstudio interface (51, 52). Fluorescence intensity profiles of single replicates are shown in Fig. S6a and c.

### Purification of the AAC(3)IV apramycin acetyltransferase.

For heterologous protein overproduction, E. coli BL21(DE3) cells containing the pAN6_aac(3)IV_CStrep plasmid were cultivated as described in “Bacterial strains and growth conditions.” Precultivation was performed in LB medium supplemented with 50 μg/mL kanamycin (LB Kan_50_), which was incubated overnight at 37°C and 120 rpm. The main culture in LB Kan_50_ medium was inoculated to an OD_600_ of 0.1 using the preculture. At an OD_600_ of 0.6, gene expression was induced using 100 μM IPTG (isopropyl-β-d-thiogalactopyranoside). Cells were harvested after additional 24 h of incubation at 20°C.

Cell harvesting and disruption were performed as described earlier (53) using buffer A (100 mM Tris-HCl [pH 8.0]) with cOmplete protease inhibitor (Roche, Basel, Switzerland) for cell disruption and buffer B (100 mM Tris-HCl, 500 mM NaCl [pH 8.0]) for purification. Purification of the Strep-tagged AAC(3)IV apramycin acetyltransferase was conducted by applying the supernatant to an equilibrated 2-mL Strep-Tactin–Sepharose column (IBA, Göttingen, Germany). After washing with 20 mL buffer B, the protein was eluted with 5 mL buffer B containing 15 mM d-desthiobiotin (Sigma-Aldrich, St. Louis, MO, USA).

After purification, the purity of the elution fractions was checked by SDS-PAGE (54) using a 4 to 20% Mini-Protean gradient gel (Bio-Rad, Munich, Germany). The protein concentration of the elution fraction was determined with the Pierce bicinchoninic acid (BCA) protein assay kit (Thermo Fisher Scientific, Waltham, MA, USA), and the elution fraction with the highest protein concentration was chosen for further use.

### *In vitro* acetylation reaction of apramycin.

Protein purification of the AAC(3)IV apramycin acetyltransferase was conducted as described above. Acetylation of apramycin was performed using a modified version of the protocol described by Magalhaes and Blanchard (21). Assay mixtures were composed of 100 μL 100 mM Tris-HCl–500 mM NaCl (pH 8.0) containing the AAC(3)IV at a concentration of 10 μg/mL, as well as 10 mM apramycin (approximately 5 mg/mL) and 10 mM acetyl-CoA sodium salt (Sigma-Aldrich, St. Louis, MO, USA). The assay mixtures were incubated at 37°C for 20 min.

### Data availability.

Raw data as well as processed tables were deposited in the GEO database under the accession number GSE171784.

10.1128/mbio.00783-22.10 VIDEO S1 Apramycin prevents cell lysis during infection of *S. venezuelae* with phage Alderaan. Time-lapse video of *S. venezuelae* ATCC 10712 carrying the plasmid pIJLK04, which was cultivated in a microfluidics system and challenged with Alderaan (10^8^ PFU/mL; flow rate, 200 nL/min) in presence and absence of 5 or 10 μg/mL apramycin. Download Movie S1, MOV file, 2 MB. Copyright © 2022 Kever et al. 2022 Kever et al. https://creativecommons.org/licenses/by/4.0/ This content is distributed under the terms of the Creative Commons Attribution 4.0 International license.
