# Supplementary material for: Aminoglycoside Antibiotics Inhibit Phage Infection by Blocking an Early Step of the Infection Cycle
Source: mBio. 2022 May 4;13(3):e00783-22. doi: 10.1128/mbio.00783-22 (PMC9239200; doi:10.1128/mbio.00783-22)
Supplement: TABLE S1 [file mbio.00783-22-s0001.docx]

**Supplementary Table S1: Aminoglycoside-modifying enzymes used in this study**

| Antibiotic | Gene | Annotation | Protein sequence | Modification |
| --- | --- | --- | --- | --- |
| Apramycin | *aac(3)IV*  *(apr)* | Aminoglycoside N(3)-acetyltransferase | VQYEWRKAELIGQLLNLGVTPGGVLLVHSSFRSVRPLEDGPLGLIEALRAALGPGGTLVMPSWSGLDDEPFDPATSPVTPDLGVVSDTFWRLPNVKRSAHPFAFAAAGPQAEQIISDPLPLPPHSPASPVARVHELDGQVLLLGVGHDANTTLHLAELMAKVPYGVPRHCTILQDGKLVRVDYLENDHCCERFALADRWLKEKSLQKEGPVGHAFARLIRSRDIVATALGQLGRDPLIFLHPPEAGCEECDAARQSIG | Acetylation of 3-amino group of the deoxystreptamine ring |
| Hygromycin | *aph(7'')-Ia* | Aminoglycoside O-phosphotransferase APH(7'')-Ia, | VTQESLLLLDRIDSDDSYASLRNDQEFWEPLARRALEELGLPVPPVLRVPGESTNPVLVGEPDPVIKLFGEHWCGPESLASESEAYAVLADAPVPVPRLLGRGELRPGTGAWPWPYLVMSRMTGTTWRSAMDGTTDRNALLALARELGRVLGRLHRVPLTGNTVLTPHSEVFPELLRERRAATVEDHRGWGYLSPRLLDRLEDWLPDVDTLLAGREPRFVHGDLHGTNIFVDLAATEVTGIVDFTDVYAGDSRYSLVQLHLNAFRGDREILAALLDGAQWKRTEDFARELLAFTFLHDFEVFEETPLDLSGFTDPEELAQFLWGPPDTAPGA | Phosphorylation of hydroxyl group at position 7'' |
| Kanamycin | *aph(3\\')-Ia* | Aminoglycoside 3'-phosphotransferase | MSHIQRETSCSRPRLNSNMDADLYGYKWARDNVGQSGATIYRLYGKPDAPELFLKHGKGSVANDVTDEMVRLNWLTEFMPLPTIKHFIRTPDDAWLLTTAIPGKTAFQVLEEYPDSGENIVDALAVFLRRLHSIPVCNCPFNSDRVFRLAQAQSRMNNGLVDASDFDDERNGWPVEQVWKEMHKLLPFSPDSVVTHGDFSLDNLIFDEGKLIGCIDVGRVGIADRYQDLAILWNCLGEFSPSLQKRLFQKYGIDNPDMNKLQFHLMLDEFF | Phosphorylation of hydroxyl group at position 3‘ |
| Spectinomycin/ Streptomycin | *aadA* | Aminoglycoside (3'') (9) adenylyltransferase | MREAVIAEVSTQLSEVVGVIERHLEPTLLAVHLYGSAVDGGLKPHSDIDLLVTVTVRLDETTRRALINDLLETSASPGESEILRAVEVTIVVHDDIIPWRYPAKRELQFGEWQRNDILAGIFEPATIDIDLAILLTKAREHSVALVGPAAEELFDPVPEQDLFEALNETLTLWNSPPDWAGDERNVVLTLSRIWYSAVTGKIAPKDVAADWAMERLPAQYQPVILEARQAYLGQEEDRLASRADQLEEFVHYVKGEITKVVGK | O-adenylation at positions 3″ and 9 |
