# Supplementary material for: Aminoglycoside Antibiotics Inhibit Phage Infection by Blocking an Early Step of the Infection Cycle
Source: mBio. 2022 May 4;13(3):e00783-22. doi: 10.1128/mbio.00783-22 (PMC9239200; doi:10.1128/mbio.00783-22)
Supplement: TABLE S2 [file mbio.00783-22-s0002.docx]

**Supplementary Table S2A: Bacterial strains used in this study**

| Strains | Genotype | Reference |
| --- | --- | --- |
| *C. glutamicum* MB001 | ATCC 13032 strain with deletion of prophages ΔCGP1 (cg1507-cg1524), ΔCGP2 (cg1746-cg1752) und ΔCGP3 (cg1890-cg2071) | ^1^ |
| *C. glutamicum* MB001 – pEKEx2a | MB001 carrying the plasmid pEKEx2a, Kan^R^ | This study |
| *C. glutamicum* MB001 – pEKEx2b | MB001 carrying the plasmid pEKEx2b, Hyg^R^ | This study |
| *C. glutamicum* MB001 – pEKEx2d | MB001 carrying the plasmid pEKEx2d, Apr^R^ | This study |
| *C. glutamicum* MB001 – pEKEx2e | MB001 carrying the plasmid pEKEx2e, Sp^R^/Sm^R^ | This study |
| *Escherichia coli* DH5α | *supE44 ΔlacU169 (f80lacZ*DM15*) hsdR17 recA1 endA1 gyrA96 thi-1 relA1* | Invitrogen |
| *Escherichia coli* ET12567/pUZ8002 | *dam-13*∷*Tn9 dcm-6 hsdM hsdR,* carrying plasmid pUZ8002 | ^2^ |
| *Escherichia coli* BL21 (DE3) | F^-^ *ompT hsdS_B_(r_B_^−^ m_B_ ^−^) gal dcm* λ(DE3) | ^3^ |
| *Escherichia coli* DSM 613 | Wild-type strain | ^4^ |
| *E. coli* DSM 613 – pEKEx2a | *E. coli* DSM 613 carrying the plasmid pEKEx2a, Kan^R^ | This study |
| *E. coli* DSM 613 – pEKEx2b | *E. coli* DSM 613 carrying the plasmid pEKEx2b, Hyg^R^ | This study |
| *E. coli* DSM 613 – pEKEx2d | *E. coli* DSM 613 carrying the plasmid pEKEx2d, Apr^R^ | This study |
| *E. coli* DSM 613 – pEKEx2e | *E. coli* DSM 613 carrying the plasmid pEKEx2e, Sp^R^/Sm^R^ | This study |
| *Escherichia coli* DSM 5695 | *F^+^ met str T1^s^ T6^s^ lambda^-^* | ^5^ |
| *E. coli* DSM 5695 – pEKEx2a | *E. coli* DSM 5695 carrying the plasmid pEKEx2a, Kan^R^ | This study |
| *E. coli* DSM 5695 – pEKEx2b | *E. coli* DSM 5695 carrying the plasmid pEKEx2b, Hyg^R^ | This study |
| *E. coli* DSM 5695 – pEKEx2d | *E. coli* DSM 5695 carrying the plasmid pEKEx2d, Apr^R^ | This study |
| *E. coli* DSM 5695 – pEKEx2e | *E. coli* DSM 5695 carrying the plasmid pEKEx2e, Sp^R^/Sm^R^ | This study |
| *Escherichia coli* DSM 4230 | *F^-^ hsdR514 (rk^-^ mk^-^) supE44 supF58 Δ(lacIZY)6 galK2 galT22 metB1 trpR55 lambda^-^* | ^6^ |
| *E. coli* DSM 4230 – pEKEx2a | *E. coli* DSM 4230 carrying the plasmid pEKEx2a, Kan^R^ | This study |
| *E. coli* DSM 4230 – pEKEx2b | *E. coli* DSM 4230 carrying the plasmid pEKEx2b, Hyg^R^ | This study |
| *E. coli* DSM 4230 – pEKEx2d | *E. coli* DSM 4230 carrying the plasmid pEKEx2d, Apr^R^ | This study |
| *E. coli* DSM 4230 – pEKEx2e | *E. coli* DSM 4230 carrying the plasmid pEKEx2e, Sp^R^/Sm^R^ | This study |
| *Escherichia coli* JW3996 | *E. coli* BW25113 Δ*lamB* | ^7^ |
| *Streptomyces venezuelae* ATCC 10712 | Wild-type strain | ^8^ |
| *S. venezuelae* ATCC 10712 – pIJLK01 | *S****.*** *venezuelae* ATCC 10712 carrying the integrative plasmid pIJLK01, Hyg^R^ | This study |
| *S. venezuelae* ATCC 10712 – pIJLK04 | *S****.*** *venezuelae* ATCC 10712 carrying the integrative plasmid pIJLK04, Apr^R^ | This study |
| *S. venezuelae* ATCC 10712 – pIJLK05 | *S****.*** *venezuelae* ATCC 10712 carrying the integrative plasmid pIJLK05, Sp^R^/Sm^R^ | This study |
| *Streptomyces coelicolor* M145 | *S. coelicolor* A3(2) lacking plasmids SCP1 and SCP2 | ^9^ |
| *S. coelicolor* M145– pIJLK01 | *S****.*** *coelicolor* M145 carrying the integrative plasmid pIJLK01, Hyg^R^ | This study |
| *S. coelicolor* M145– pIJLK04 | *S****.*** *coelicolor* M145 carrying the integrative plasmid pIJLK04, Apr^R^ | This study |
| *S. coelicolor* M145– pIJLK05 | *S****.*** *coelicolor* M145 carrying the integrative plasmid pIJLK05, Sp^R^/Sm^R^ | This study |

**Supplementary Table S2B: Phages used in this study**

| **Phage** | **Host organism** | **Lifestyle** | **Family** | **Genome** | **State of injected genome**^13,14^ | **Reference** |
| --- | --- | --- | --- | --- | --- | --- |
| **Alderaan** | *S. venezuelae* ATCC 10712 | Virulent | *Siphoviridae* | dsDNA | Linear with terminal redundancy | ^15^ |
| **Coruscant** | *S. venezuelae* ATCC 10712 | Virulent | *Siphoviridae* | dsDNA | Linear with terminal repeats | ^15^ |
| **Dagobah** | *S. coelicolor* M145 | Temperate | *Siphoviridae* | dsDNA | Linear with terminal repeats | ^15^ |
| **Endor1** | *S. coelicolor* M145 | Temperate | *Siphoviridae* | dsDNA | Linear with terminal redundancy | ^15^ |
| **Endor2** | *S. coelicolor* M145 | Temperate | *Siphoviridae* | dsDNA | Linear with terminal redundancy | ^15^ |
| **CL31** | *C. glutamicum* MB001 | Temperate | *Siphoviridae* | dsDNA | Linear with cohesive ends | ^16^ |
| **Spe2** | *C. glutamicum* ATCC 13032 | Virulent | *Siphoviridae* | dsDNA | Unknown | This study,  DSM110582 |
| **T4** | *E. coli* B (DSM613) | Virulent | *Myoviridae* | dsDNA | Linear with terminal redundancy | DSM4505 |
| **T5** | *E. coli* B (DSM613) | Virulent | *Siphoviridae* | dsDNA | Linear with terminal redundancy | DSM16353 |
| **T6** | *E. coli* B (DSM613) | Virulent | *Myoviridae* | dsDNA | Linear with terminal repeats | DSM4622 |
| **T7** | *E. coli* B (DSM613) | Virulent | *Podoviridae* | dsDNA | Linear with terminal repeats | DSM4623 |
| **M13** | *E. coli* W1485 (DSM5695) | Chronic infection | *Inoviridae* | ssDNA | Circular (+) strand | DSM13976 |
| **fd** | *E. coli* W1485 (DSM5695) | Chronic infection | *Inoviridae* | ssDNA | Circular (+) strand | DSM4498 |
| **MS2** | *E. coli* W1485 (DSM5695) | Virulent | *Leviviridae* | ssRNA | Linear, bound to the maturation protein ^17^ | DSM13767 |
| **Lambda (λ)** | *E. coli* LE392 (DSM4230) | Temperate | *Siphoviridae* | dsDNA | Linear with cohesive ends | DSM4499 |

**Supplementary Table S2C: Plasmids used in this study.** Insert DNA was amplified using the listed oligonucleotides (compare Supplementary File 5). Linearization of vector DNA was conducted with the indicated restriction enzyme and plasmids were constructed using Gibson assembly. Sequencing was performed by Eurofins Genomics (Ebersberg, Germany) with the sequencing oligonucleotides listed.

| Plasmids | Characteristics | | | | | | Reference |
| --- | --- | --- | --- | --- | --- | --- | --- |
| pIJ10257 | Hyg^R^; Cloning vector for the conjugal transfer of DNA from *E. coli* to *Streptomyces spp.;* (constitutive promoter *ermE**; Integration at the ΦBT1 attachment site) | | | | | | ^18^ |
| pEKEx2 | Kan^R^; *C. glutamicum*/ *E. coli* shuttle vector for regulated gene expression; *P_tac_, lacI^q^*, pBL1 oriV*_C.g._*, pUC18 oriV*_E.c._* | | | | | | ^19^ |
| pIJ773 | pBluescript II SK(+)-based plasmid containing the apramycin resistance cassette  flanked by FRT (FLP recognition target) recombination sites | | | | | | ^20^ |
| pCDFduet-1 | Sp^R^/Sm^R^; *E. coli* vector for coexpression of two target genes; P_T7_, *lacI*, CloDF13 ori, T7 terminator | | | | | | Novagen |
| pUZ8002 | Kan^R^; RK2 derivative with nontransmissible oriT | | | | | | ^21^ |
| pAN6 | Kan^R^.; *E. coli* vector for regulated gene expression; derivative of pEKEx2 (*P*_tac_, *lacI*^q^, pBL1 *oriV_C.g_*_._, pUC18 *oriV_E.c_*_._) | | | | | | ^22^ |
| Plasmids | **Characteristics** | **Template** | **Primer** | **Vector** | **Restriction enzyme** | **Sequencing**  **primer** | **Reference** |
| pIJLK01 | Hyg^R^; Derivative of pIJ10257 with additional restrictions sites Bst1107I (upstream) and StuI (downstream) of the *aph(7'')-Ia* gene allowing exchanging of the antibiotic cassette | pIJ10257 | 1 + 2 3 + 4 5 + 6 | pIJ10257 | KpnI;  PvuII | 25 - 28 | This study |
| pIJLK04 | Apr^R^; Derivative of pIJLK01 with *aph(7'')-Ia* exchanged for *aac(3)IV* (apramycin resistance gene) | pIJ773 | 7 + 8 | pIJLK01 | Bst1107I; StuI | 28 | This study |
| pIJLK05 | Sp^R^/Sm^R^; Derivative of pIJLK01 with *aph(7'')-Ia* exchanged for *aadA* (spectinomycin/ streptomycin resistance gene) | pCDFduet-1 | 9 + 10 | pIJLK01 | Bst1107I; StuI | 28 | This study |
| pEKEx2a | Kan^R^; Derivative of pEKEx2 with additional restrictions sites Bst1107I (upstream) and NotI (downstream) of the *aphA1* gene allowing exchanging of the antibiotic cassette | pEKEx2 | 11 + 12  13 + 14  15 + 16 | pEKEx2 | SapI;  StuI | 29 - 32 | This study |
| pEKEx2b | Hyg^R^; Derivative of pEKEx2a with *aphA1* exchanged for *aph(7'')-Ia* (hygromycin resistance gene) | pIJ10257 | 17 + 18 | pEKEx2a | Bst1107I; NotI | 31 + 32 | This study |
| pEKEx2d | Apr^R^; Derivative of pEKEx2a with *aphA1* exchanged for *aac(3)IV* (apramycin resistance gene) | pIJ773 | 19 + 20 | pEKEx2a | Bst1107I; NotI | 31 + 32 | This study |
| pEKEx2e | Sp^R^/Sm^R^; Derivative of pEKEx2a with *aphA1*exchanged for *aadA* (spectinomycin/ streptomycin resistance gene) | pCDFduet-1 | 21 + 22 | pEKEx2a | Bst1107I; NotI | 31 + 32 | This study |
| pAN6_  aac(3)IV_Cstrep | Kan^R^; Derivative of pAN6 with *aac(3)IV* fused to a C-terminal Strep-tag | pIJ773 | 23 + 24 | pAN6_  CStrep | NdeI;  NheI | 33 + 34 | This study |

Supplementary Table S2D: Oligonucleotides used in this study

| No. | Oligonucleotide name | Sequence (5' - 3') |
| --- | --- | --- |
| Construction of plasmids | | |
| 1 | pIJ10257_RE1_1_fw | TGCTCGGGTCGGGCTGGTACCAGTGAGCGTTTTTCAACCTCAG |
| 2 | pIJ10257_RE1_1_rv | GATTCTTGTGTCACGTATACAGCGGACCTCTATTCACAGGG |
| 3 | pIJ10257_RE1_2_fw | AATAGAGGTCCGCTGTATACGTGACACAAGAATCCCTGTTACTTCTCG |
| 4 | pIJ10257_RE2_2_rv | CGGGCGGCCCGGGGCGAGGCCTTCAGGCGCCGGGGG |
| 5 | pIJ10257_RE2_3_fw | CCCCCGGCGCCTGAAGGCCTCGCCCCGGGCCGC |
| 6 | pIJ10257_RE2_3_rv | GAAACCTGTCGTGCCAGCTGCATTAATGAATCGGCCAACGCGC |
| 7 | pIJLK04_aac(3)IV_fw | TGAATAGAGGTCCGCTGTATACGTGCAATACGAATGGCGAAAAG |
| 8 | pIJLK04_aac(3)IV_rv | GGCGGCCCGGGGCGAGGCCTTCAGCCAATCGACTGGCG |
| 9 | pIJLK05_aadA_fw | TGAATAGAGGTCCGCTGTATACATGAGGGAAGCGGTGATCG |
| 10 | pIJLK05_aadA_rv | GCGGCCCGGGGCGAGGCCTTTATTTGCCGACTACCTTGGTGAT |
| 11 | pEKEx2_RE1_1_fw | GCGGTTTGCGTATTGGGCGCTCT |
| 12 | pEKEx2_RE1_1_rv | ATGGCTCATGTATACAACACCCCTTGTATTACTGTTTATGTAAGCAGAC |
| 13 | pEKEx2_RE1_2_fw | GGGGTGTTGTATACATGAGCCATATTCAACGGGAAACGTCT |
| 14 | pEKEx2_RE2_2_rv | TTCTGAGCGGCCGCTTAGAAAAACTCATCGAGCATCAAATGAAAC |
| 15 | pEKEx2_RE2_3_fw | TTTCTAAGCGGCCGCTCAGAATTGGTTAATTGGTTGTAACA |
| 16 | pEKEx2_RE2_3_rv | CGTGAAGAAGGTGTTGCTGACTC |
| 17 | pEKEx2b_hygR_fw | ATACAAGGGGTGTTGTATACGTGACACAAGAATCCCTGTTACTTCTC |
| 18 | pEKEx2b_hygR_rv | AATTAACCAATTCTGAGCGGCCGCTCAGGCGCCGGGGGC |
| 19 | pEKEx2d_aac(3)IV_fw | ATACAAGGGGTGTTGTATACGTGCAATACGAATGGCGAAAAG |
| 20 | pEKEx2d_aac(3)IV_rv | ACCAATTCTGAGCGGCCGCTCAGCCAATCGACTGGCGAG |
| 21 | pEKEx2e_aadA_fw | ACAAGGGGTGTTGTATACATGAGGGAAGCGGTGATCG |
| 22 | pEKEx2e_aadA_rv | ACCAATTCTGAGCGGCCGCTTATTTGCCGACTACCTTGGTGATC |
| 23 | pAN6_aac(3)IV_Cstrep_ fw | CCTGCAGAAGGAGATATACATATGATGTCATCAGCGGTGGAG |
| 24 | pAN6_aac(3)IV_CStrep_rv | TGTGGGTGGGACCAGCTAGCGCCAATCGACTGGCGAGC |
| Sequencing primer | | |
| 25 | pIJLK01_seq_fw_1 | GATCAACCGCGACTAGCATC |
| 26 | pIJLK01_seq_fw_2 | CCGGTGATCAAGCTGTTC |
| 27 | pIJLK01_seq_fw_3 | TTTCTGCGCGTAATCTGCTG |
| 28 | pIJLK0x_seq_fw_4 | CGTAGAGATTGGCGATCCC |
| 29 | pEKEx2a_seq_fw | TTCCAGTCGGGAAACCTGTC |
| 30 | pEKEx2a_seq_rv | TCGCGAGCCCATTTATACCC |
| 31 | pEKEx2x_seq_fw | GGAAAGCCACGTTGTGTCTC |
| 32 | pEKEx2x_seq_rv | GCCTCGTGAAGAAGGTGTTG |
| 33 | pAN6_seq_ Cstrep_fw | CGGCGTTTCACTTCTGAGTTCGGC |
| 34 | pAN6_seq_ Cstrep_rv | GATATGACCATGATTACGCC |
| qPCR primer | | |
| 35 | qPCR_*atpD*_Sv_fw | TGTTCGAGACCGGCCTGAAG |
| 36 | qPCR_*atpD*_Sv_rv | AGACACCGTCGTGCAGCTTG |
| 37 | qPCR_Alderaan_HQ601_00028_fw | CTCGGCTATCCGATCATCC |
| 38 | qPCR_Alderaan_HQ601_00028_rv | TTGGTTGCGGTTGATGGAC |

**References**

1 Baumgart, M. *et al.* Construction of a prophage-free variant of *Corynebacterium glutamicum* ATCC 13032 for use as a platform strain for basic research and industrial biotechnology. *Appl Environ Microbiol* **79**, 6006-6015, doi:10.1128/AEM.01634-13 (2013).

2 MacNeil, D. J. *et al.* Analysis of *Streptomyces avermitilis* genes required for avermectin biosynthesis utilizing a novel integration vector. *Gene* **111**, 61-68, doi:10.1016/0378-1119(92)90603-M (1992).

3 Studier, F. W. & Moffatt, B. A. Use of Bacteriophage T7 RNA Polymerase to Direct Selective High-level Expression of Cloned Genes *J Mol Biol* **189**, 113-130, doi:10.1016/0022-2836(86)90385-2 (1986).

4 Luria, S. E., Delbrück, M. & Anderson, T. F. Electron microscope studies of bacterial viruses. *Journal of Bacteriology* **46**, 57-77, doi:10.1128/JB.46.1.57-77.1943 (1943).

5 Lederberg, E. M. & Lederberg, J. Genetic Studies of Lysogenicity in *Escherichia Coli*. *Genetics* **38**, 51-64 (1953).

6 Murray, N. E., Brammar, W. J. & Murray, K. Lambdoid Phages that Simplify the Recovery of in vitro Recombinants. *Mol Gen Genet.* **150**, 53-61, doi:10.1007/BF02425325. (1977).

7 Baba, T. *et al.* Construction of *Escherichia coli* K-12 in-frame, single-gene knockout mutants: the Keio collection. *Mol Syst Biol* **2**, 2006 0008, doi:10.1038/msb4100050 (2006).

8 Ehrlich, J., Gottlieb, D., Burkholder, P. R., Anderson, L. E. & Pridham, T. G. *Streptomyces venezuelae*, n. sp., the source of chloromycetin. *Journal of Bacteriology* **56**, 467-477, doi:10.1128/jb.56.4.467-477.1948 (1948).

9 Bentley, S. D. *et al.* Complete genome sequence of the model actinomycete *Streptomyces coelicolor* A3(2). *Nature* **417**, 141-147, doi:10.1038/417141a (2002).

10 Higgins, C. E. & Kastner, R. E. Nebramycin, a new broad-spectrum antibiotic complex. II. Description of *Streptomyces tenebrarius*. *Antimicrobial agents and chemotherapy* **7**, 324-331 (1967).

11 Lv, M. *et al.* Characterization of a C3 Deoxygenation Pathway Reveals a Key Branch Point in Aminoglycoside Biosynthesis. *Journal of the American Chemical Society* **138**, 6427-6435, doi:10.1021/jacs.6b02221 (2016).

12 Zhang, Q., Chi, H. T., Wu, L., Deng, Z. & Yu, Y. Two Cryptic Self-Resistance Mechanisms in *Streptomyces tenebrarius* Reveal Insights into the Biosynthesis of Apramycin. *Angewandte Chemie (International ed. in English)* **60**, 8990-8996, doi:10.1002/anie.202100687 (2021).

13 Kronheim, S. *et al.* A chemical defence against phage infection. *Nature* **564**, 283-286, doi:10.1038/s41586-018-0767-x (2018).

14 Casjens, S. R. & Gilcrease, E. B. Determining DNA packaging strategy by analysis of the termini of the chromosomes in tailed-bacteriophage virions. *Methods in molecular biology (Clifton, N.J.)* **502**, 91-111, doi:10.1007/978-1-60327-565-1_7 (2009).

15 Hardy, A., Sharma, V., Kever, L. & Frunzke, J. Genome sequence and characterization of five bacteriophages infecting *Streptomyces coelicolor* and *Streptomyces venezuelae*: Alderaan, Coruscant, Dagobah, Endor1 and Endor2. *Viruses* **12**, 1065, doi:10.3390/v12101065 (2020).

16 Hünnefeld, M. *et al.* Genome Sequence of the Bacteriophage CL31 and Interaction with the Host Strain *Corynebacterium glutamicum* ATCC 13032. *Viruses* **13**, 495, doi:10.3390/v13030495 (2021).

17 Harb, L. *et al.* ssRNA phage penetration triggers detachment of the F-pilus. *Proc Natl Acad Sci U S A* **117**, 25751-25758, doi:10.1073/pnas.2011901117 (2020).

18 Hong, H. J., Hutchings, M. I., Hill, L. M. & Buttner, M. J. The role of the novel Fem protein VanK in vancomycin resistance in *Streptomyces coelicolor*. *J Biol Chem* **280**, 13055-13061, doi:10.1074/jbc.M413801200 (2005).

19 Eikmanns, B. J., Kleinertz, E., Liehl, W. & Sahm, H. A family of *Corynebacterium glutamicum/Escherichia coli* shuttle vectors for cloning, controlled gene expression, and promoter probing. *Gene* **102**, 93-98, doi:10.1016/0378-1119(91)90545-M (1991).

20 Gust, B., Challis, G. L., Fowler, K., Kieser, T. & Chater, K. F. PCR-targeted *Streptomyces* gene replacement identifies a protein domain needed for biosynthesis of the sesquiterpene soil odor geosmin. *PNAS* **100**, 1541–1546, doi:10.1073/pnas.0337542100 (2003).

21 Paget, M. S. B., Chamberlin, L., Atrih, A., Foster, S. J. & Buttner, M. J. Evidence that the Extracytoplasmic Function Sigma Factor s^E^ Is Required for Normal Cell Wall Structure in *Streptomyces coelicolor* A3(2). *Journal of Bacteriology* **181**, 204-211, doi:10.1128/JB.181.1.204-211.1999 (1999).

22 Frunzke, J., Engels, V., Hasenbein, S., Gätgens, C. & Bott, M. Co-ordinated regulation of gluconate catabolism and glucose uptake in *Corynebacterium glutamicum* by two functionally equivalent transcriptional regulators, GntR1 and GntR2. *Mol Microbiol* **67**, 305-322, doi:10.1111/j.1365-2958.2007.06020.x (2008).
