# Supplementary figures and images for: Aminoglycoside Antibiotics Inhibit Phage Infection by Blocking an Early Step of the Infection Cycle
Source: mBio. 2022 May 4;13(3):e00783-22. doi: 10.1128/mbio.00783-22 (PMC9239200; doi:10.1128/mbio.00783-22)

**a**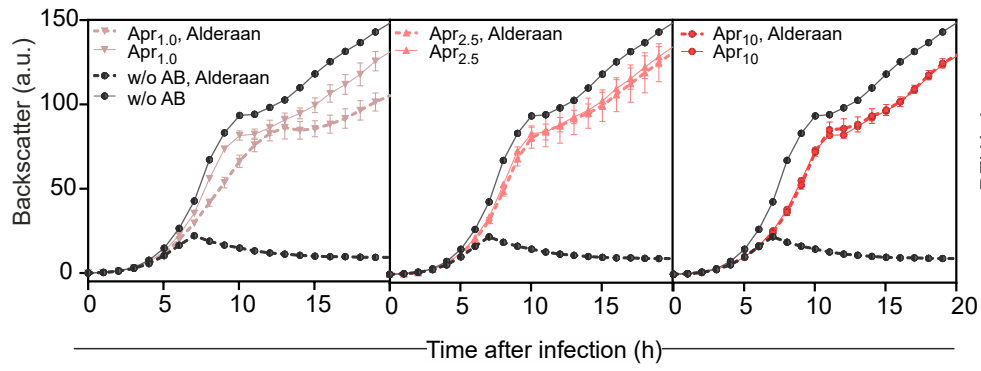**b**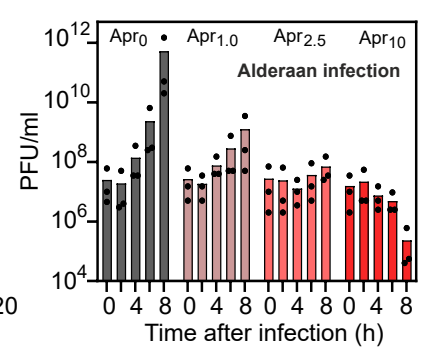

Supplement: FIG S1 [file mbio.00783-22-s0004.pdf]

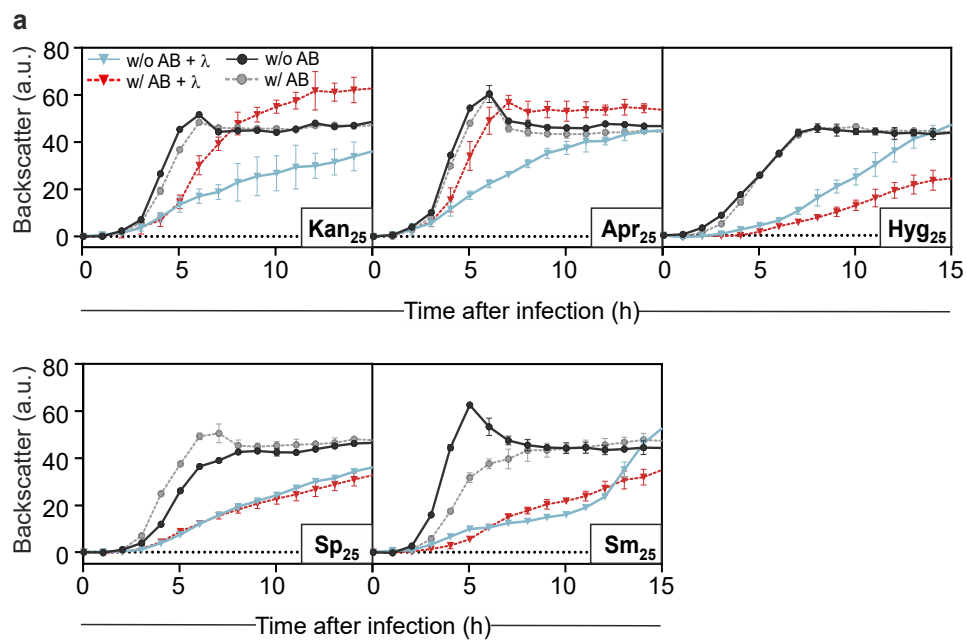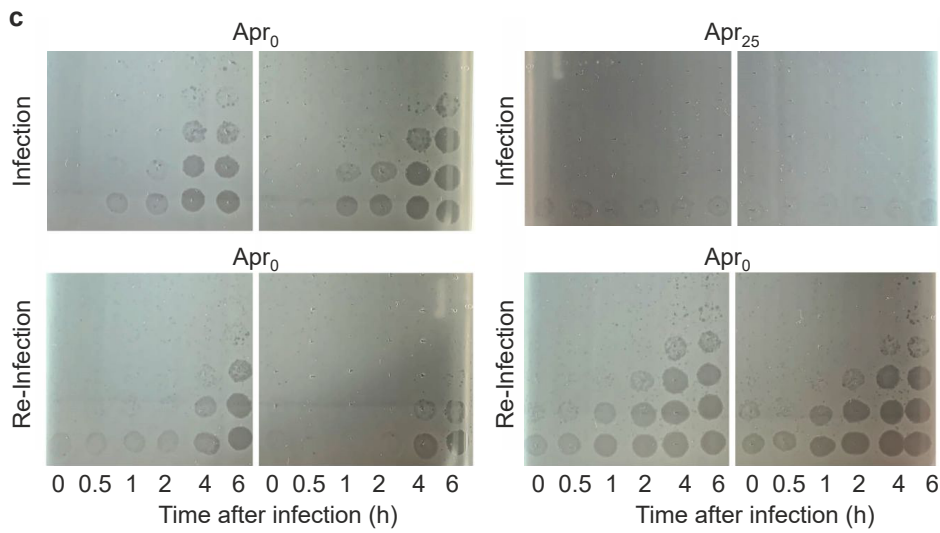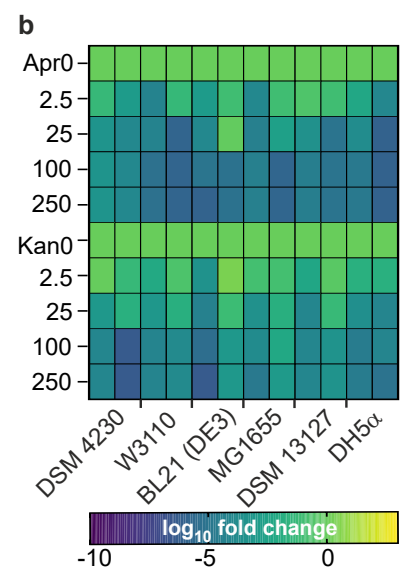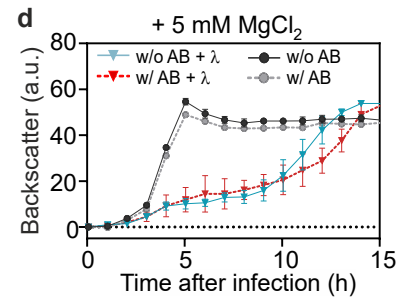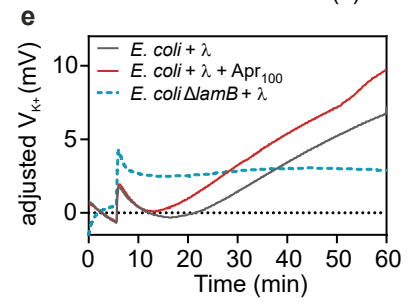

Supplement: FIG S2 [file mbio.00783-22-s0005.pdf]

**a**

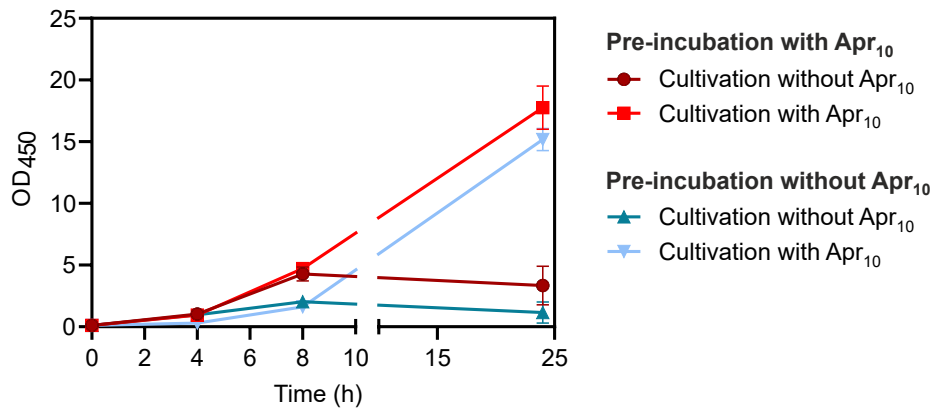

**b**

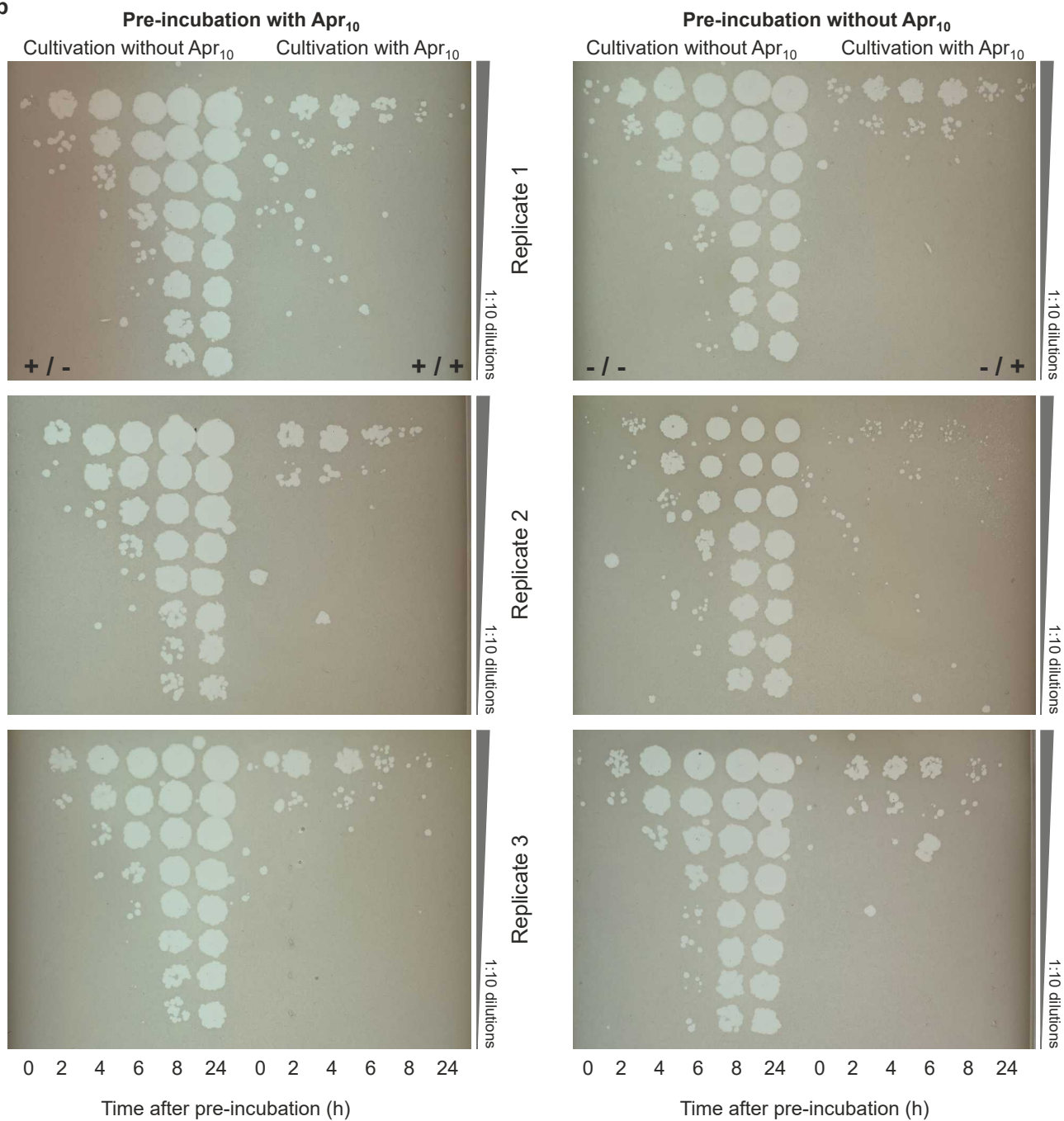

Supplement: FIG S3 [file mbio.00783-22-s0006.pdf]

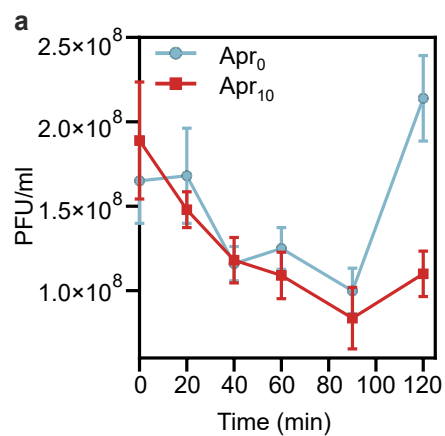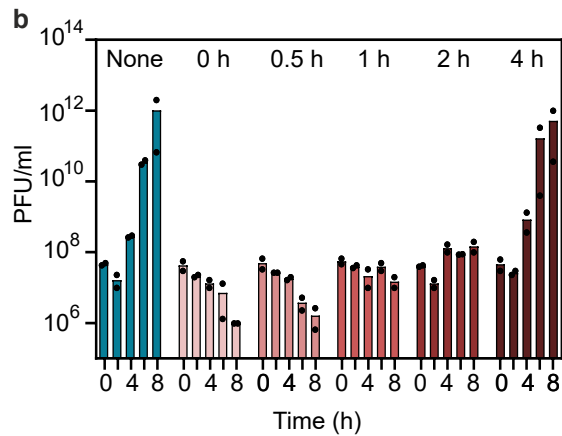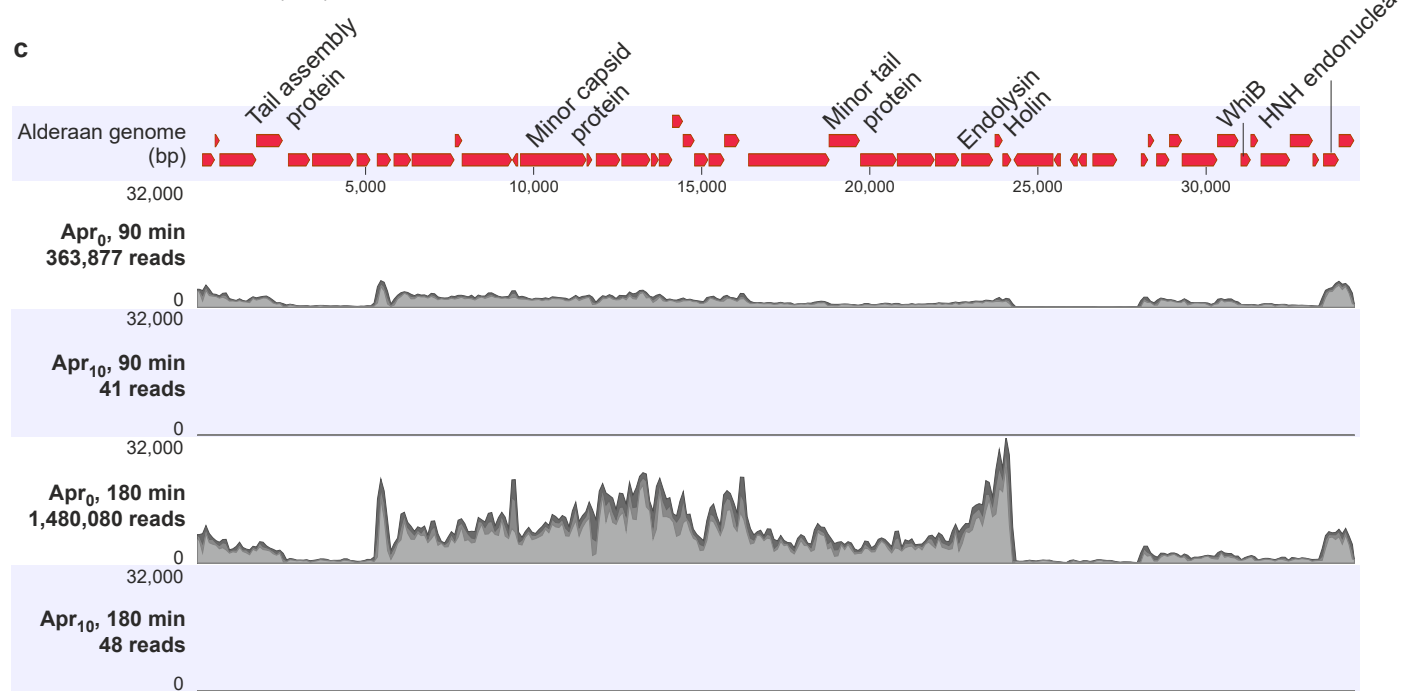

Supplement: FIG S4 [file mbio.00783-22-s0007.pdf]

# Pre-incubation of Alderaan in GYM Apr<sub>x</sub>

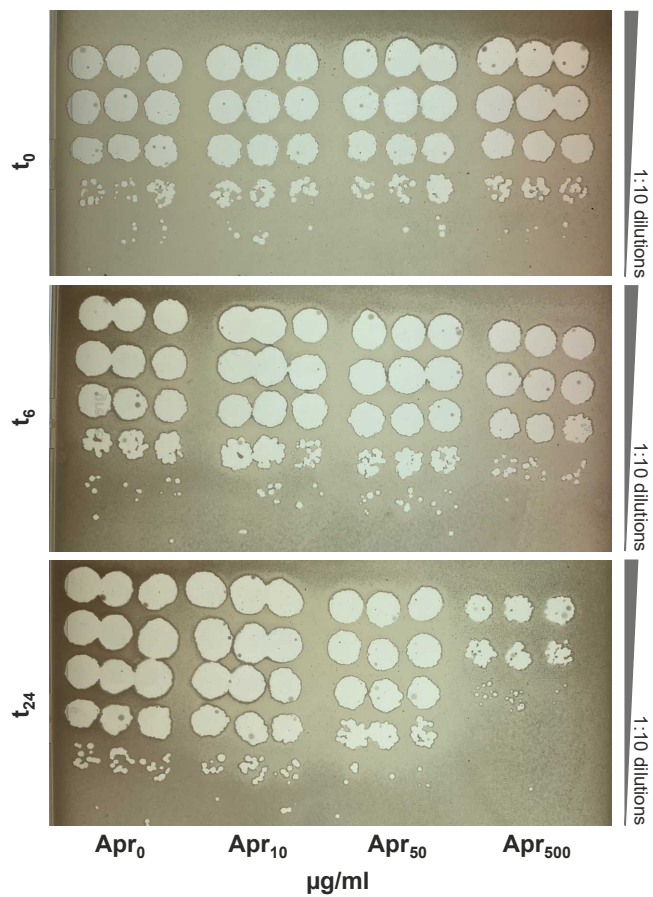

Supplement: FIG S5 [file mbio.00783-22-s0008.pdf]

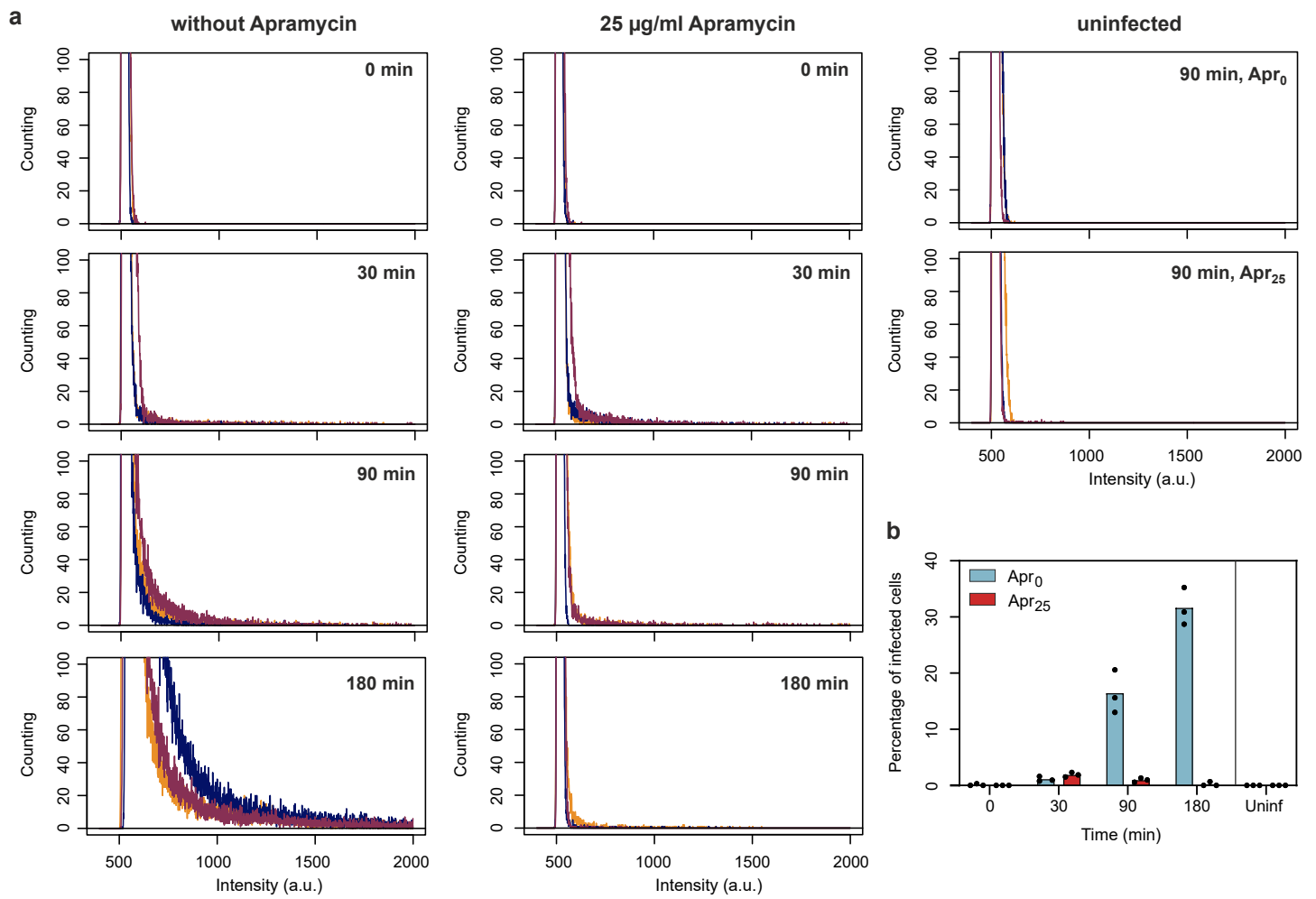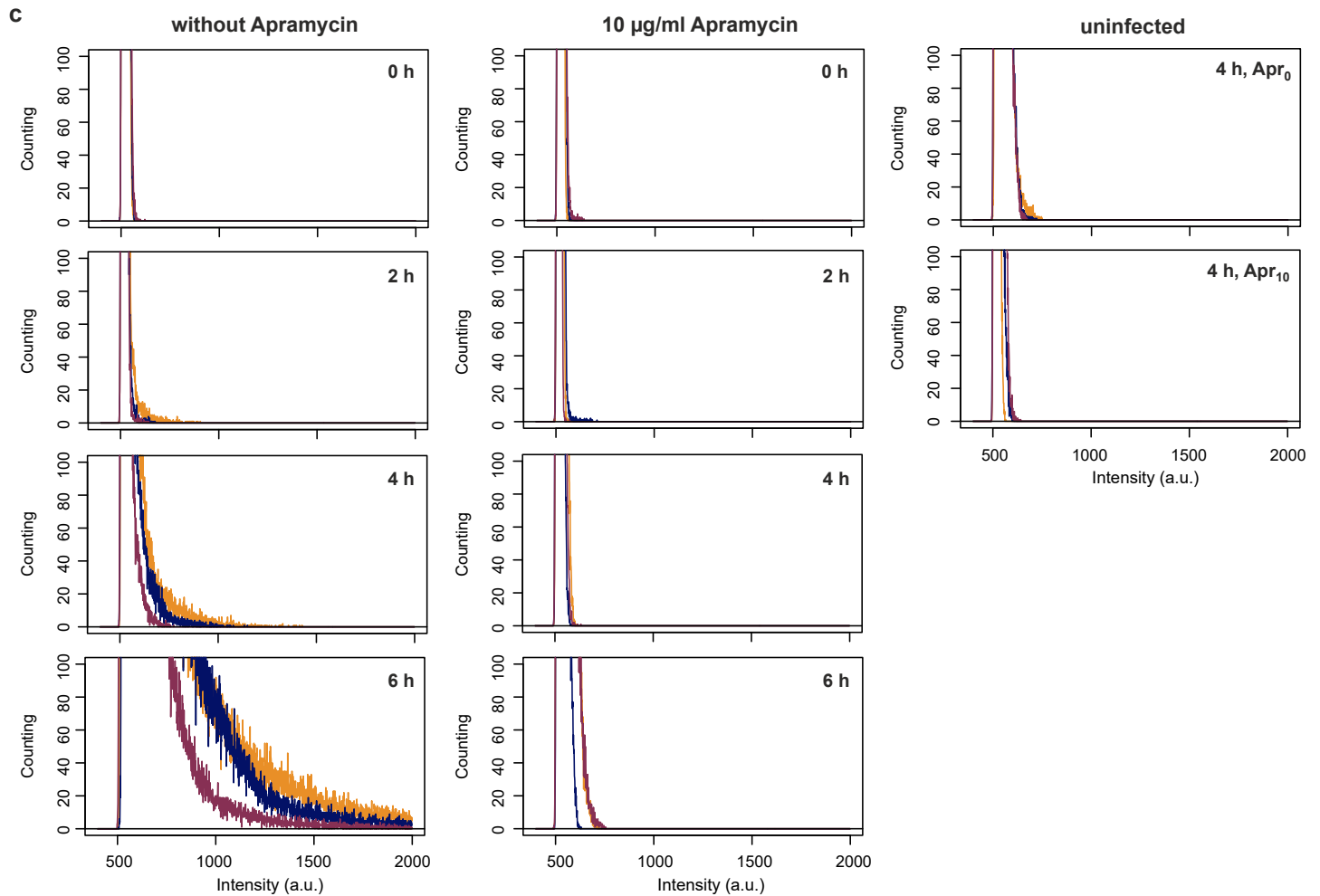

Supplement: FIG S6 [file mbio.00783-22-s0009.pdf]
