# Supplementary material for: Aminoglycoside Antibiotics Inhibit Phage Infection by Blocking an Early Step of the Infection Cycle
Source: mBio. 2022 May 4;13(3):e00783-22. doi: 10.1128/mbio.00783-22 (PMC9239200; doi:10.1128/mbio.00783-22)
Supplement: TABLE S3 [file mbio.00783-22-s0003.docx]

**Supplementary Table S3: Polynucleotides used for phage targeting direct-geneFISH**

| No. | Sequence (5' - 3') |
| --- | --- |
| Gene probes for phage targeting direct-geneFISH with Alderaan infecting *S. venezuelae* | |
| 1 | acggcgatcagcacccaggacaggacgacgtctttgtgtcgcacccaccatggcggtctcggtctcggctcttccatcaactttcccccagttcggcaacagtcgacatcgtatggagagagggggtgagcccgtgcccattcaccccggcatggtcgagcccctcgccgaacgcacccgcgatctctacgccgccgccg |
| 2 | tacgcggacgccgaggacgcctgtaacggctacctcctgaacaagaaggccaaggcggacggcatcaacccggccgccctgttcagcggcccagcccgtatcgcgtacgcccgagcgtcggacgagctgaaagagtggtgggccgaacacggtcgcctaacgcaggcggagttcatcgagcaggtcaccggcaaggctca |
| 3 | cgccgacagcaacggccgtcacgagcacgtcaccaactacgacgtcgccacggcctcccccaccaccccgtaaggaggtccgcccatggcgggtaacagcaggtccatcgacgcgcgcggatggctcttcgaggtcaaggacaccgacgccagcaccgagacgtggctcccgatcgccggtctcaactcctggtcgtact |
| 4 | cggcgcgtacgaggaggacgtcatgcagcgcggcgcctccatcaccctggagggtcagtaccgcatcgacaagacgaccaaggcccgcgacgtgggacaggcgtacatcgatgaggaatggacgccgcgtctcggcatcgactcgcacaaccagatccgctaccggcacgagacgcagtccgcatgggcgatctgggacg |
| 5 | cgtagacgtcgccgaagacctcttggacacgttcgccgagtatccgacggagttccgtcagctcggcctcgacgccgagacggcgatggggctgctctcccagggactccaaggcggtgcgcgcgacgccgatacggtcgccgacgcgttgaaggaattcacgctcatggctcagggcatgggcgagtcaactgcggagt |
| 6 | gccgtgtgggccggggccatcgtcgtcggtcagtgggtcctcatggccacacaggccctcatgcaggccgcccgcatggccgccgcgtggctcatcgccatgggcccgattggcctgctcatcgctgccgtggtcggtctcgtcgtcctgatcatcaagtattgggatgacatcgtcgcagccaccacgaaggcgtggga |
| 7 | ggccgccggcgactacgtcgagatgaccattttcagcggcgccgccctgtccggcattccgtccagctacagccgggcgtcgttggtctggcagggccccgcgtgagcggcatgtaccgcgtcgttctgtgcgacctgcgatccgaccaagtcctcgacatccttcccgcgcagggcatcaagtgcgacgactacatcgg |
| 8 | ccgcgacgttcgactcgtacctcgcgcaccggctactcaaggacgggtggaccgggaacggggtcgaccaactcgacatcgcccgtcagatcgtcgactgggtccagtcgaccgagggcggcaacatcggcatcgaactggactggtcgcagacatccggagtgctccgcgaccgggcgtactcccgctacgacctgtac |
| 9 | gtcgtgcgcgacgtgctcgaccaactcgccaacgtcgagaacgggttcgagtggcgcgtacgtacgtaccgcgatgcgtccggccgccgcgtgaagagcttgcagctcggctatccgatcatccggagcagccgtaccgagttggttctctcctccccgggcccggtcatcgactaccggatgcccgaggacggcacctc |
| 10 | gcgccaccgcgcagcttcagttccgcgtgaacggaaccatcgtggcgaccggcacggcgggtcaaccgctccttgccaccttcgccatcccgtcgtacgcgttcggcatgaacgccgagttcgagctacaggcccgcgtgtccagcggcaccggaaccgcctacgcccagacccgctacctgtacggcttccagtcctaa |
| 11 | ggcgcggtgctcggcgcggtgctcggcacggtcgtcgtggcgctcatcggctgctctcctccttgcggggcgtgctccggaatggctccaccttgacaccccggtccggacgtatctgcccccaaagcgaggacagttgagacgtctctcgctagggtcgaataaggcccaaaagtccgggcagagggggttgacagtga |
| 12 | aaccgcgcggacgctgcacgggcgctgggagtggatgaggaaatgatctggccgaaggcggtgcaggaccgcgtaaaggtcggcggcgaccgggagatcctccgcacttacccataccgctcggcgtgcccctccaacgtgtgggcggacctcgctgccggcgccgagcacgagctgtttctcgccgggtatacgaacta |
| 13 | gcgaggtgacccggcagcgcgaggtaatcgaaggcgttccgctgtcggtttccacgcgcattcggatcacgctcgatgagttggcgcggctcgggtcggtcgagggtgtcgaggcccggctgagcgctgccgaggatgccgtaaatcacgtgagcctgtcggtattccgattcgacgaggaggccctcgtaacgcctcat |
| 14 | tgcggcggcacgttgacggcgggatgttcgaccgcttcgcagagcacgccgaagagctgtgggagcgggccgtgcccgtgacgtcgtagacgacgaacggccccgcctcccagacagggacgcggggccgtcgcgtttccgcaggtcaagcaaccattccgtatcgctcccctttaggtccactgtcgtggggtgttgga |
| 15 | gacgcgagtgtctgtgactgcttcgcccctgaggagccggccgacggtcgaggcgctgatgccgctatctgcggcaaagcgggagcgccccccgctgcgtaggccggtgacgtcgtatccgcgtctcacgagttcctgggagagccatgcggcgaacgccgagcgcgtcgcgtcggtcttgttttctgccatggcagaaa |
| 16 | ccgcgcggcgagaagggcacgcacaccgtgctccggatgctgtaccgcatctacggcccggccggcgactgcctcgccgtcaccatcccgggcgaggccatggacacggccgacaagagcaccaacaaggcgatgtcggccgcgctcaagtacatgctctttcaggtgttcatgatccccgtggacgcccgcagcatcga |
| 17 | ggtgatcccgcccgcgttcaccccggaggacggcacctagcccccctcgatagggggagccggatcagccccacatccgctatctttctgtctcgacagatacatgcctgtcgagacagaaccgcgagcgcgcgcgctcagcacgtagagcaggaggaccgccccgtgaacacccccgagcgcttcgccgccaaggtcga |
| Gene probes for phage targeting direct-geneFISH with λ infecting *E. coli* | |
| 1 | agcagtatcttaaatttggcgacaaagagacgccgtttggcctcaaatggacgccggatgacccctccagcgtgttttatctctgcgagcataatgcctgcgtcatccgccagcaggagctggactttactgatgcccgttatatctgcgaaaagaccgggatctggacccgtgatggcattctctggttttcgtcatccggtgaagagattgagccacctgacagtgtgacctttcacatctggacagcgtacagcccgttcaccacctgggtgcagattgtcaaagactggatgaaaa |
| 2 | gcaggacaacgtattcgatgtgttatctgaaagtactgatgaacggtgcggtgatttatgatggcgcggcgaacgaggcggtacaggtgttctcccgtattgttgacatgccagcgggtcggggaaacgtgatcctgacgttcacgcttacgtccacacggcattcggcagatattccgccgtatacgtttgccagcgatgtgcaggttatggtgattaagaaacaggcgctgggcatcagcgtggtctgagtgtgttacagaggttcgtccgggaacgggcgttttattataaaacagt |
| 3 | agattattatgggccgccacgacgatgaacagacgctgctgcgtgtggatgaggccatcaataaaacctatacccgccggaatggtgcagaaatgtcgatatcccgtatctgctgggatactggcgggattgacccgaccattgtgtatgaacgctcgaaaaaacatgggctgttccgggtgatccccattaaaggggcatccgtctacggaaagccggtggccagcatgccacgtaagcgaaacaaaaacggggtttaccttaccgaaatcggtacggataccgcgaaagagcagattt |
| 4 | caattttgtcccactccctgcctctgtcatcacgatactgtgatgccatggtgtccgacttatgcccgagaagatgttgagcaaacttatcgcttatctgcttctcatagagtcttgcagacaaactgcgcaactcgtgaaaggtaggcggatccccttcgaaggaaagacctgatgcttttcgtgcgcgcataaaataccttgatactgtgccggatgaaagcggttcgcgacgagtagatgcaattatggtttctccgccaagaatctctttgcatttatcaagtgtttccttcattg |
| 5 | tgctcgacataaagatatccatctacgatatcagaccacttcatttcgcataaatcaccaactcgttgcccggtaacaacagccagttccattgcaagtctgagccaacatggtgatgattctgctgcttgataaattttcaggtattcgtcagccgtaagtcttgatctccttacctctgattttgctgcgcgagtggcagcgacatggtttgttgttatatggccttcagctattgcctctcggaatgcatcgctcagtgttgatctgattaacttggctgacgccgccttgccctcg |
| 6 | aactcaatgttggcctgtatagcttcagtgattgcgattcgcctgtctctgcctaatccaaactctttacccgtccttgggtccctgtagcagtaatatccattgtttcttatataaaggttagggggtaaatcccggcgctcatgacttcgccttcttcccatttctgatcctcttcaaaaggccacctgttactggtcgatttaagtcaacctttaccgctgattcgtggaacagatactctcttccatccttaaccggaggtgggaatatcctgcattcccgaacccatcgacgaac |
| 7 | tgtttcaaggcttcttggacgtcgctggcgtgcgttccactcctgaagtgtcaagtacatcgcaaagtctccgcaattacacgcaagaaaaaaccgccatcaggcggcttggtgttctttcagttcttcaattcgaatattggttacgtctgcatgtgctatctgcgcccatatcatccagtggtcgtagcagtcgttgatgttctccgcttcgataactctgttgaatggctctccattccattctcctgtgactcggaagtgcatttatcatctccataaaacaaaacccgccgtagc |
| 8 | actcaacccgatgtttgagtacggtcatcatctgacactacagactctggcatcgctgtgaagacgacgcgaaattcagcattttcacaagcgttatcttttacaaaaccgatctcactctcctttgatgcgaatgccagcgtcagacatcatatgcagatactcacctgcatcctgaacccattgacctccaaccccgtaatagcgatgcgtaatgatgtcgatagttactaacgggtcttgttcgattaactgccgcagaaactcttccaggtcaccagtgcagtgcttgataacagg |
| 9 | gttcatccagcagttccagcacaatcgatggtgttaccaattcatggaaaaggtctgcgtcaaatccccagtcgtcatgcattgcctgctctgccgcttcacgcagtgcctgagagttaatttcgctcacttcgaacctctctgtttactgataagttccagatcctcctggcaacttgcacaagtccgacaaccctgaacgaccaggcgtcttcgttcatctatcggatcgccacactcacaacaatgagtggcagatatagcctggtggttcaggcggcgcatttttattgctgtgtt |
| 10 | tgagggtgaatgcgaataataaaaaaggagcctgtagctccctgatgattttgcttttcatgttcatcgttccttaaagacgccgtttaacatgccgattgccaggcttaaatgagtcggtgtgaatcccatcagcgttaccgtttcgcggtgcttcttcagtacgctacggcaaatgtcatcgacgtttttatccggaaactgctgtctggctttttttgatttcagaattagcctgacgggcaatgctgcgaagggcgttttcctgctgaggtgtcattgaacaagtcccatgtcggc |
| 11 | aggtaaacgggcatttcagttcaaggccgttgccgtcactgcataaaccatcgggagagcaggcggtacgcatactttcgtcgcgatagatgatcggggattcagtaacattcacgccggaagtgaattcaaacagggttctggcgtcgttctcgtactgttttccccaggccagtgctttagcgttaacttccggagccacaccggtgcaaacctcagcaagcagggtgtggaagtaggacattttcatgtcaggccacttctttccggagcggggttttgctatcacgttgtgaactt |
| 12 | tgatgacgccgagccgtaatttgtgccacgcatcatccccctgttcgacagctctcacatcgatcccggtacgctgcaggataatgtccggtgtcatgctgccaccttctgctctgcggctttctgtttcaggaatccaagagcttttactgcttcggcctgtgtcagttctgacgatgcacgaatgtcgcggcgaaatatctgggaacagagcggcaataagtcgtcatcccatgttttatccagggcgatcagcagagtgttaatctcctgcatggtttcatcgttaaccggagtgat |
| 13 | tcgcgttccggctgacgttctgcagtgtatgcagtattttcgacaatgcgctcggcttcatccttgtcatagataccagcaaatccgaaggccagacgggcacactgaatcatggctttatgacgtaacatccgtttgggatgcgactgccacggccccgtgatttctctgccttcgcgagttttgaatggttcgcggcggcattcatccatccattcggtaacgcagatcggatgattacggtccttgcggtaaatccggcatgtacaggattcattgtcctgctcaaagtccatgcca |
| 14 | tcaaactgctggttttcattgatgatgcgggaccagccatcaacgcccaccaccggaacgatgccattctgcttatcaggaaaggcgtaaatttctttcgtccacggattaaggccgtactggttggcaacgatcagtaatgcgatgaactgcgcatcgctggcatcacctttaaatgccgtctggcgaagagtggtgatcagttcctgtgggtcgacagaatccatgccgacacgttcagccagcttcccagccagcgttgcgagtgcagtactcattcgttttatacctctgaatcaa |
| 15 | tatcaacctggtggtgagcaatggtttcaaccatgtaccggatgtgttctgccatgcgctcctgaaactcaacatcgtcatcaaacgcacgggtaatggattttttgctggccccgtggcgttgcaaatgatcgatgcatagcgattcaaacaggtgctggggcaggcctttttccatgtcgtctgccagttctgcctctttctcttcacgggcgagctgctggtagtgacgcgcccagctctgagcctcaagacgatcctgaatgtaataagcgttcatggctgaactcctgaaatagc |
| 16 | gataaagccaaggccaatatctaagtaactagataagaggaatcgattttcccttaattttctggcgtccactgcatgttatgccgcgttcgccaggcttgctgtaccatgtgcgctgattcttgcgctcaatacgttgcaggttgctttcaatctgtttgtggtattcagccagcactgtaaggtctatcggatttagtgcgctttctactcgtgatttcggtttgcgattcagcgagagaatagggcggttaactggttttgcgcttaccccaaccaacaggggatttgctgctttcc |
| 17 | agcctgtttctctgcgcgacgttcgcggcggcgtgtttgtgcatccatctggattctcctgtcagttagctttggtggtgtgtggcagttgtagtcctgaacgaaaaccccccgcgattggcacattggcagctaatccggaatcgcacttacggccaatgcttcgtttcgtatcacacaccccaaagccttctgctttgaatgctgcccttcttcagggcttaatttttaagagcgtcaccttcatggtggtcagtgcgtcctgctgatgtgctcagtatcaccgccagtggtatttat |
| 18 | tactatgttatgttctgaggggagtgaaaattcccctaattcgatgaagattcttgctcaattgttatcagctatgcgccgaccagaacaccttgccgatcagccaaacgtctcttcaggccactgactagcgataactttccccacaacggaacaactctcattgcatgggatcattgggtactgtgggtttagtggttgtaaaaacacctgaccgctatccctgatcagtttcttgaaggtaaactcatcacccccaagtctggctatgcagaaatcacctggctcaacagcctgctc |
| 19 | tatttgcatacattcaatcaattgttatctaaggaaatacttacatatggttcgtgcaaacaaacgcaacgaggctctacgaatcgagagtgcgttgcttaacaaaatcgcaatgcttggaactgagaagacagcggaagctgtgggcgttgataagtcgcagatcagcaggtggaagagggactggattccaaagttctcaatgctgcttgctgttcttgaatggggggtcgttgacgacgacatggctcgattggcgcgacaagttgctgcgattctcaccaataaaaaacgcccggc |
| 20 | tcaagcagcaaggcggcatgtttggaccaaataaaaacatctcagaatggtgcatccctcaaaacgagggaaaatcccctaaaacgagggataaaacatccctcaaattgggggattgctatccctcaaaacagggggacacaaaagacactattacaaaagaaaaaagaaaagattattcgtcagagaattctggcgaatcctctgaccagccagaaaacgacctttctgtggtgaaaccggatgctgcaattcagagcggcagcaagtgggggacagcagaagacctgaccgccgcag |
| 21 | ataagtggacccaactcgaaatcaaccgtaacaagcaacaggcaggcgtgacagccagcaaaccaaaactcgacctgacaaacacagactggatttacggggtggatctatgaaaaacatcgccgcacagatggttaactttgaccgtgagcagatgcgtcggatcgccaacaacatgccggaacagtacgacgaaaagccgcaggtacagcaggtagcgcagatcatcaacggtgtgttcagccagttactggcaactttcccggcgagcctggctaaccgtgaccagaacgaagtgaa |
| 22 | tggcggtatatggagttaaaagatgaccatctacattactgagctaataacaggcctgctggtaatcgcaggcctttttatttgggggagagggaagtcatgaaaaaactaacctttgaaattcgatctccagcacatcagcaaaacgctattcacgcagtacagcaaatccttccagacccaaccaaaccaatcgtagtaaccattcaggaacgcaaccgcagcttagaccaaaacaggaagctatgggcctgcttaggtgacgtctctcgtcaggttgaatggcatggtcgctggctg |
| 23 | gatgcagaaagctggaagtgtgtgtttaccgcagcattaaagcagcaggatgttgttcctaaccttgccgggaatggctttgtggtaataggccagtcaaccagcaggatgcgtgtaggcgaatttgcggagctattagagcttatacaggcattcggtacagagcgtggcgttaagtggtcagacgaagcgagactggctctggagtggaaagcgagatggggagacagggctgcatgataaatgtcgttagtttctccggtggcaggacgtcagcatatttgctctggctaatggagc |
| 24 | ccatttcgggcgagggaattacaccacgtggattggcatcagagctgatgaaccgaagcggctaaagccaaagcctggaatcagatatcttgctgaactgtcagactttgagaaggaagatatcctcgcatggtggaagcaacaaccattcgatttgcaaataccggaacatctcggtaactgcatattctgcattaaaaaatcaacgcaaaaaatcggacttgcctgcaaagatgaggagggattgcagcgtgtttttaatgaggtcatcacgggatcccatgtgcgtgacggacatcg |
| 25 | ggaaacgccaaaggagattatgtaccgaggaagaatgtcgctggacggtatcgcgaaaatgtattcagaaaatgattatcaagccctgtatcaggacatggtacgagctaaaagattcgataccggctcttgttctgagtcatgcgaaatatttggagggcagcttgatttcgacttcgggagggaagctgcatgatgcgatgttatcggtgcggtgaatgcaaagaagataaccgcttccgaccaaatcaaccttactggaatcgatggtgtctccggtgtgaaagaacaccaacaggg |
| 26 | gtgttaccactaccgcaggaaaaggaggacgtgtggcgagacagcgacgaagtatcaccgacataatctgcgaaaactgcaaataccttccaacgaaacgcaccagaaataaacccaagccaatcccaaaagaatctgacgtaaaaaccttcaactacacggctcacctgtgggatatccggtggctaagacgtcgtgcgaggaaaacaaggtgattgaccaaaatcgaagttacgaacaagaaagcgtcgagcgagctttaacgtgcgctaactgcggtcagaagctgcatgtgctgga |
| 27 | gcgcagaactgatgagcgatccgaatagctcgatgcacgaggaagaagatgatggctaaaccagcgcgaagacgatgtaaaaacgatgaatgccgggaatggtttcaccctgcattcgctaatcagtggtggtgctctccagagtgtggaaccaagatagcactcgaacgacgaagtaaagaacgcgaaaaagcggaaaaagcagcagagaagaaacgacgacgagaggagcagaaacagaaagataaacttaagattcgaaaactcgccttaaagccccgcagttactggattaaacaa |
| 28 | ccaacaagccgtaaacgccttcatcagagaaagagaccgcgacttaccatgtatctcgtgcggaacgctcacgtctgctcagtgggatgccggacattaccggacaactgctgcggcacctcaactccgatttaatgaacgcaatattcacaagcaatgcgtggtgtgcaaccagcacaaaagcggaaatctcgttccgtatcgcgtcgaactgattagccgcatcgggcaggaagcagtagacgaaatcgaatcaaaccataaccgccatcgctggactatcgaagagtgcaaggcgat |
| 29 | tgttatctgccacgccgattatccctttgacgaatacgagtttggaaagccagttgatcatcagcaggtaatctggaaccgcgaacgaatcagcaactcacaaaacgggatcgtgaaagaaatcaaaggcgcggacacgttcatctttggtcatacgccagcagtgaaaccactcaagtttgccaaccaaatgtatatcgataccggcgcagtgttctgcggaaacctaacattgattcaggtacagggagaaggcgcatgagactcgaaagcgtagctaaatttcattcgccaaaaagc |
| 30 | cagagattgccatggtacaggccgtgcggttgatattgccaaaacagagctgtgggggagagttgtcgagaaagagtgcggaagatgcaaaggcgtcggctattcaaggatgccagcaagcgcagcatatcgcgctgtgacgatgctaatcccaaaccttacccaacccacctggtcacgcactgttaagccgctgtatgacgctctggtggtgcaatgccacaaagaagagtcaatcgcagacaacattttgaatgcggtcacacgttagcagcatgattgccacggatggcaacatat |
| 31 | tgaataaaattgggtaaatttgactcaacgatgggttaattcgctcgttgtggtagtgagatgaaaagaggcggcgcttactaccgattccgcctagttggtcacttcgacgtatcgtctggaactccaaccatcgcaggcagagaggtctgcaaaatgcaatcccgaaacagttcgcaggtaatagttagagcctgcataacggtttcgggattttttatatctgcacaacaggtaagagcattgagtcgataatcgtgaagagtcggcgagcctggttagccagtgctctttccgttg |
| 32 | tgctgaattaagcgaataccggaagcagaaccggatcaccaaatgcgtacaggcgtcatcgccgcccagcaacagcacaacccaaactgagccgtagccactgtctgtcctgaattcattagtaatagttacgctgcggccttttacacatgaccttcgtgaaagcgggtggcaggaggtcgcgctaacaacctcctgccgttttgcccgtgcatatcggtcacgaacaaatctgattactaaacacagtagcctggatttgttctatcagtaatcgaccttattcctaattaaatagag |
| 33 | caaatccccttattgggggtaagacatgaagatgccagaaaaacatgacctgttggccgccattctcgcggcaaaggaacaaggcatcggggcaatccttgcgtttgcaatggcgtaccttcgcggcagatataatggcggtgcgtttacaaaaacagtaatcgacgcaacgatgtgcgccattatcgcctggttcattcgtgaccttctcgacttcgccggactaagtagcaatctcgcttatataacgagcgtgtttatcggctacatcggtactgactcgattggttcgcttatcaa |
| 34 | atcatggttatgacgtcattgtaggcggagagctatttactgattactccgatcaccctcgcaaacttgtcacgctaaacccaaaactcaaatcaacaggcgccggacgctaccagcttctttcccgttggtgggatgcctaccgcaagcagcttggcctgaaagacttctctccgaaaagtcaggacgctgtggcattgcagcagattaaggagcgtggcgctttacctatgattgatcgtggtgatatccgtcaggcaatcgaccgttgcagcaatatctgggcttcactgccgggcg |
| 35 | gataaaacaaaagccaccgtgtcggtcagtggtatgaccatcaccgtgaacggcgttgctgcaggcaaggtcaacattccggttgtatccggtaatggtgagtttgctgcggttgcagaaattaccgtcaccgccagttaatccggagagtcagcgatgttcctgaaaaccgaatcatttgaacataacggtgtgaccgtcacgctttctgaactgtcagccctgcagcgcattgagcatctcgccctgatgaaacggcaggcagaacaggcggagtcagacagcaaccggaagtttact |
